# Bibliometric and Visualized Analysis of China's Smart Grid Research 2008–2018

**DOI:** 10.3389/frma.2020.551147

**Published:** 2020-10-28

**Authors:** Cheng Wang, Tao Lv, Xu Deng

**Affiliations:** ^1^School of Management, China University of Mining and Technology, Xuzhou, China; ^2^School of Foreign Languages, Huaiyin Normal University, Huai'an, China

**Keywords:** smart grid (SG), bibliometrics, co-authorship, co-occurrence, VOSviewer, alluvial diagram, China

## Abstract

Smart grid (SG) offers great advantages in renewable energy integration and has become a popular trend of modern power development recently; meanwhile China is the second most prolific country using SG. Hence the purpose of this study is to get access to the research status, development, and trends of SG in China based on the 3,558 published papers obtained from the WOS core library and application of the bibliometric method and visualization analysis software VOSviewer and alluvial diagrams. The results consequently demonstrate some valuable insights. Firstly, the volume of publications in China's SG is on the rise, and the cooperation between countries and institutions is getting closer. Besides, the research hotspots have obvious interdisciplinary characteristics. Taking into consideration the impact of the information and communication field on SG, the major current research hotspots include wireless sensor network (WSN), internet of things (IoT), smart meter, big data, and security. Taking into consideration the impact of SG on traditional power systems, the main hotspots cover demand response, micro-grid, distributed generation, and electric vehicle (EV). Furthermore, China's SG research shows a trend from a single theme to diversified development. The research themes during 2010–2018 have deepened with most studies focusing on the traditional power system. The findings of this paper provide some enlightenment on China's SG research, which can present scholars with an overview of the macro perspective, help them understand the latest development of the SG field in China and offer useful guidance for future research in this subject as well.

## Introduction

The integration of large-scale renewable energy in power systems is inevitable, but large-scale renewable energy is intermittent and variable, which has a great impact on the power grid. Smart grid (SG) with the ability of rapid response and self-repair has become an important solution to the above problems, as SG can promote the use of clean energy, improve the efficiency of power generation and energy utilization, improve the transmission efficiency of power grids, and improve the energy efficiency of terminals (Yuan and Hu, 2011). Consequently, SG has become a recent developmental trend for the world power grid, which can improve the efficiency and reliability of the power grid, reduce peak demand (El-Hawary, 2017), realize automatic control, and self-repair (Gungor et al., 2011).

SG is not only a single technology but also a series of new technologies and institutional innovations that can make the grid more efficient, cleaner, and smarter. Yu et al. define SG in China as “*an integration of renewable energy, new materials, advanced equipment, information technology, control technology, and energy storage technology, which can realize digital management, intelligent decision making, and interactive transactions of electricity generation, transmission, deployment, usage, and storage*” (Yu et al., 2012). SG has several new functionalities: self-healing, motivating the consumer, resisting attack, increasing power quality, accommodating all generation and storage options, enabling electrical markets, optimizing assets, and operating efficiently (Baumeister, 2010). In recent years, SG has attracted more and more attention, and the number of related papers published has increased. So far, more than 20,000 related papers have been published in different journals.

On the basis of qualitative analysis, many scholars have published reviews on SG. Technology development is the primary condition for the development of SG, which mainly involves wireless sensor technology (Mahmood et al., 2015), communication technology (Usman and Shami, 2013; Kabalci, 2016), artificial intelligence technology (Zhang et al., 2018), big data technology (Tu et al., 2017), and internet of things technology (Hossain et al., 2019). Institutional innovations are another important area. Along with technological innovation, developed and developing countries face similar challenges in the SG area, and national incentives and national energy resources limit the development of SG (Ponce-Jara et al., 2017). The pioneers of developing countries in SG are China, India, and Brazil (Fadaeenejad et al., 2014). Yu et al. gave a comprehensive overview of China's SG development, obstacles and barriers, and policy prospects (Yu et al., 2012). Yuan et al. analyzed the policy, pilot projects, achievements, and barriers of developing SG in China, and found that the lack of national strategy and the current industrial structure of the power industry were obstacles to its development (Yuan et al., 2014).

Although these existing reviews are beneficial for scholars to understand the SG development, they have merely taken a qualitative approach to review the content and subject matter of published literature. More knowledge, therefore, can be gained by quantitatively analyzing the existing literature and exploring and tracking the evolution of a large number of published work.

At present, a large number of scholars have used bibliometric methods to quantitatively visualize the landscape and the evolution of various scientific research fields (e.g., Montoya et al., 2014; Guo et al., 2019; Merigó et al., 2019; Shi and Liu, 2019). These studies are of great help to our quantitative analysis of SG research in China. For example, 10,938 journal articles, and 144 books on SG from ScienceDirect from 2008 to 2015 were reviewed and discussed the features, functionalities, and characteristics of SG (Tuballa and Abundo, 2016). However, this paper only briefly analyzes the annual publication quantity and technical classification of literature, and subjectively selects some literature for analysis. Coincidentally, Hossain et al. searched relevant literature on SG from databases such as Elsevier, Springer, Taylor & Francis, and Wiley for analysis, and determined the role of SG in renewable energy (Hossain et al., 2016). This paper also searched the literature in SG from different databases and only selected part of the literature for categorization and analysis.

The above two works of literature, analyzed from a literature perspective, play an important role in the SG field. However, there are still some defects, mainly in the following aspects: First of all, these reviews only selected a few representative papers. Secondly, the author selects the literature subjectively according to his own experience. Thirdly, existing review articles rarely cover the research hotspots, cooperative networks, and development trends of SG. However, there are currently still too few bibliometric papers in the SG field.

Quantitative visualization analysis of the SG field in China is very useful because it can supplement and verify qualitative reviews (Zhu et al., 2019). Compared with qualitative reviews, quantitative reviews can elucidate the status quo and development within the SG field in China from a macro perspective and provide an objective and intuitive overview. Besides, with the help of visualization tools, the landscape, and evolutionary patterns of China's SG can be more intuitively displayed. Therefore, it is important and timely to conduct a quantitative review of China's SG.

In this review, we attempt to quantify the landscape and development trajectory of SG research in China and discover the current research frontiers. We reviewed 11 years of published research (2008–2018) from the WOS Core library and used the visualization tool VOSviewer to detect, quantify, and visualize the current status and evolution of SG research in China.

Our bibliometric review has the following possible contributions. Firstly, it provides a new way to discover partnerships through co-authorship and is able to show it visually. Secondly, the evolution of the frontier of SG research in China is quantitatively tracked by stages. Thirdly, this review provides an overview of SG research in China from a macro perspective. Therefore, this review can provide scholars with a systematic understanding of the status quo, research frontiers, and future trends of SG research in China, and thus promote the development of SG research in the future.

The present study aimed to answer the following research questions of China's SG:

1. What are the publication, citation status, and trend of smart grid literature? This will help researchers identify SG trends and predict future patterns in the field.
2. Which are the most influential institutions in China's SG field and what are the differences in their research hotspots? How is China's cooperation in the SG field? This will help the researcher identify the subject and potential research collaborators.
3. What are the most influential papers and journals? This will help researchers to consider which journals to choose to publish their manuscripts in within the SG field, potentially affecting future citations of their literature. On the other hand, most influential papers will help researchers and practitioners gain access to the literature that needs the most attention in the field. It will be beneficial for researchers to find research directions and methods.
4. What are China's research hotspots in the SG field? How do research hotspots evolve? This will help researchers to understand the research direction and development trend of SG in China from a global perspective, and point out the direction for future research.

To solve the above research questions, this paper is organized as follows: In section Methodology, we will introduce the research methods, including the database selection, retrieval strategy, bibliometric methods, and data cleaning. A general overview of the SG field in China will be presented in section Basic Features in SG of China, including annual changes in publications and citations, the most published Chinese institutions and influences, the most published journals, and key articles ranked by citations. Section Visualization Analysis displays a visual presentation of keyword co-occurrence and discovers research hotspots and their evolution process in three stages. In the last section, we will present the conclusions and limitations.

## Methodology

**Figure 1:**
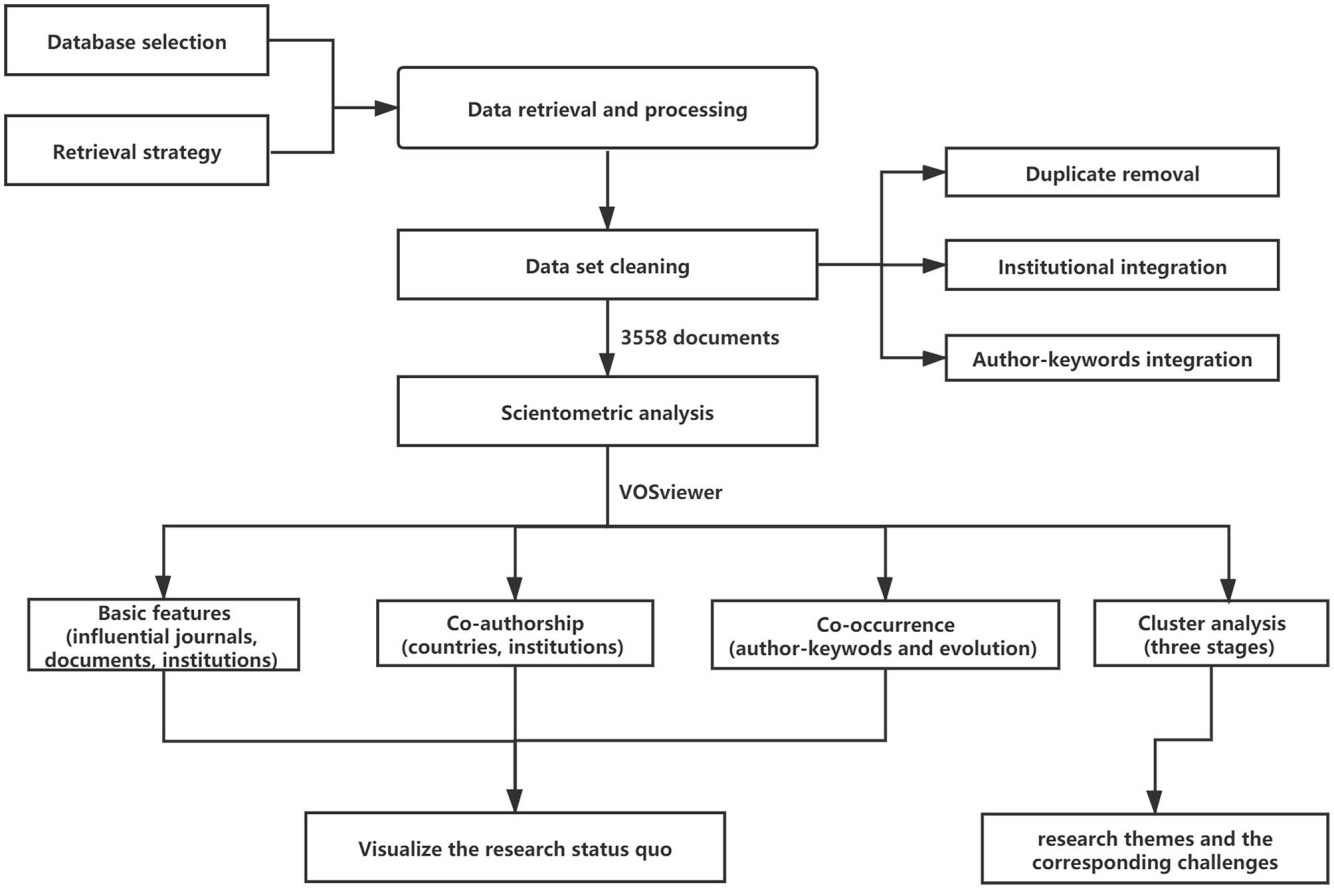
Outline of research design.

This paper provides a systematic review of scientometric analysis in SG of China. Figure 1 shows the research design of this study.

### Data Collection and Processing

Before the bibliometric analysis, it is necessary to establish a data set containing citation information. Currently, the citation databases include Scopus, ISI Web of Science (WOS), and Google Scholar. Olawumi et al. compared the advantages and disadvantages of the three databases (Olawumi et al., 2017). This paper finally selects the Science Citation Index Science (SCIE), Conference Proceedings Citation Index-Science (CPCI-S) and Social Sciences Citation Index (SSCI), which are in the core library of the WOS database. The WOS records were chosen as they contain the most comprehensive and influential journals, which have scientific robustness. Therefore, they have become the choice of many scholars for literature measurement (Rahman et al., 2017; Olawumi and Chan, 2018; Yu and He, 2020).

The search strategy adopted to retrieve the paper data on China's SG was as follows:

*TS* = (“smart grid^*^” OR “smartgrid^*^”*). Document Type: Article OR Proceedings Paper OR Review. Timespan* = *2008–2018. Databases: Science Citation Index Expanded (SCIE), Conference Proceedings Citation Index – Science (CPCI-S), Social Sciences Citation Index (SSCI). Country/Region: PEOPLES R CHINA. Search time: 2019-11-14 (SCIE and CPCI-S), 2020-03-20 (SSCI)*. *TS refers to topic research in Web of Science*.

Before we can analyze the data, we must clean the sample we received from the WOS (Mulet-Forteza et al., 2019). In the co-authorship analysis, countries that appear in different names but choose to be recognized as a single country have been unified, such as “Scotland” and “England” were united as the “United Kingdom.” “Taiwan” and “Hong Kong” were united as “People R China.” Also, the organization's name is unified. For example, “NCEPU” and “N CHINA ELECT POWER UNIV” are uniformly modified to “North China Elect Power University.” Furthermore, subordinate agencies of the State Grid Corporation of China will be integrated into the State Grid Corporation of China. For example, the names of “Guangdong POWER Grid Corporation,” “Jiangsu POWER Grid Corporation,” and “CHINA POWER SCI RES INS” will be replaced by “State Grid Corporation of China.”

Finally, in the co-occurrence of author-keywords, First of all, for the lack of author keywords, the necessary supplements were made against the original literature. Besides, different expressions of keywords have been integrated, such as unifying “smart grids,” “smart grid,” and “smart-grids” into “smart grid”; “V2G” and ”Vehicle-to-grid” have been unified into “Vehicle-to-grid (V2G).” “Demand response,” “DR” unified into “demand response (DR).”

### Scientometric Analysis Methods

In this article, we use a variety of bibliometric methods to analyze the acquired data set. Firstly, the number of publications (TP) is used to detect the quantified productivity (Ding et al., 2014), meanwhile, the number of citations (TC) is used to measure influence (Goran, 2010). “Citations per document” (TC/TP) shows the average impact per paper. Another bibliographic method is h-index, which means h number of papers published in a journal, or by an organization, have at least h citations (Hirsch, 2005). Some other common methods include the most productive institutions, journals, the most cited articles, average number of authors per article (AN/TP), average number of references per article (RN/TC), and impact factor (IF) (Garfield, 1983).

Finally, we use VOSviewer software (see www.vosviewer.com) to visualize the graphical mapping of the bibliographic data. VOSviewer can construct bibliometric networks based on data from WOS, Scopus, Dimensions, and PubMed files, or reference manager files (i.e., RIS, EndNote, and RefWorks files). It uses distance-based maps to construct a co-authorship map, co-occurrence map, citation, bibliographic coupling, and co-citation map (van Eck and Waltman, 2010). In this article, co-authorship was used for country and institution cooperation analysis. Since the early 1980s, co-authorship has been operating as a proxy for research cooperation (Subramanyam, 1983). Co-occurrence was used for author-keywords analysis (He, 1999).

There are two counting methods in VOSviewer: full counting and fractional counting. Perianes-Rodriguez et al. have compared the two methods of counting, they think that in many cases, the difference is relatively limited. There may not be a conclusion from the literature on network analysis to produce fundamental influence, especially when the conclusion is based on the analysis of the datasets. Using full counting or fractional counting, there is no essential difference between the results obtained (Perianes-Rodriguez et al., 2016). Therefore, full counting was chosen as the counting method in this paper.

## Basic Features in SG of China

We found that there were 20,195 research papers about SG around the world, among the highest were the United States with 4,457 and China with 3,558. China publishes 324 articles every year on average, so it is meaningful to conduct a global analysis on the SG field in China. The term “Chinese scholar” in this paper refers to a scholar from a Chinese institution where the author published the document.

### Annual Publications and Growth Trend

As set out in Table 1, the papers published by Chinese scholars before 2009 were no more than 10 per year, but after the year 2010 the number of publications increased significantly. Then there was a small decline in 2015, followed by a slow increase and a steady trend in 2016. Therefore, the research literature in the SG field of China can be divided into three stages: the embryonic stage before 2009, the developmental stage from 2010 to 2014, and the stable stage from 2015 to 2018.

**Table 1:** Chinese scholars' publications characteristics on SG from 2008 to 2018.

**Years**	**TP**	**AN**	**AN/TP**	**TC**	**TC/TP**	**RN**	**RN/TP**
2008	1	4	4	0	0	5	5
2009	8	39	5	15	2	145	18
2010	91	337	4	759	8	1,068	12
2011	140	508	4	1,466	10	1,812	13
2012	288	1,080	4	4,832	17	4,934	17
2013	354	1,390	4	7,949	22	6,574	19
2014	488	1,975	4	5,376	11	8,847	18
2015	476	2,084	4	4,679	10	10,473	22
2016	579	2,439	4	4,379	8	12,998	22
2017	558	2,504	4	4,164	7	15,019	27
2018	575	2,640	5	2,290	4	17,791	31
Average	323.5	1363.6	4.1	3264.5	9.1	7242.4	18.5
Total	3,558	15,000	–	35,909	–	79,666	–

*TP, total publication; AN, author number; TC, total citation; RN, reference number*.

**Figure 2:**
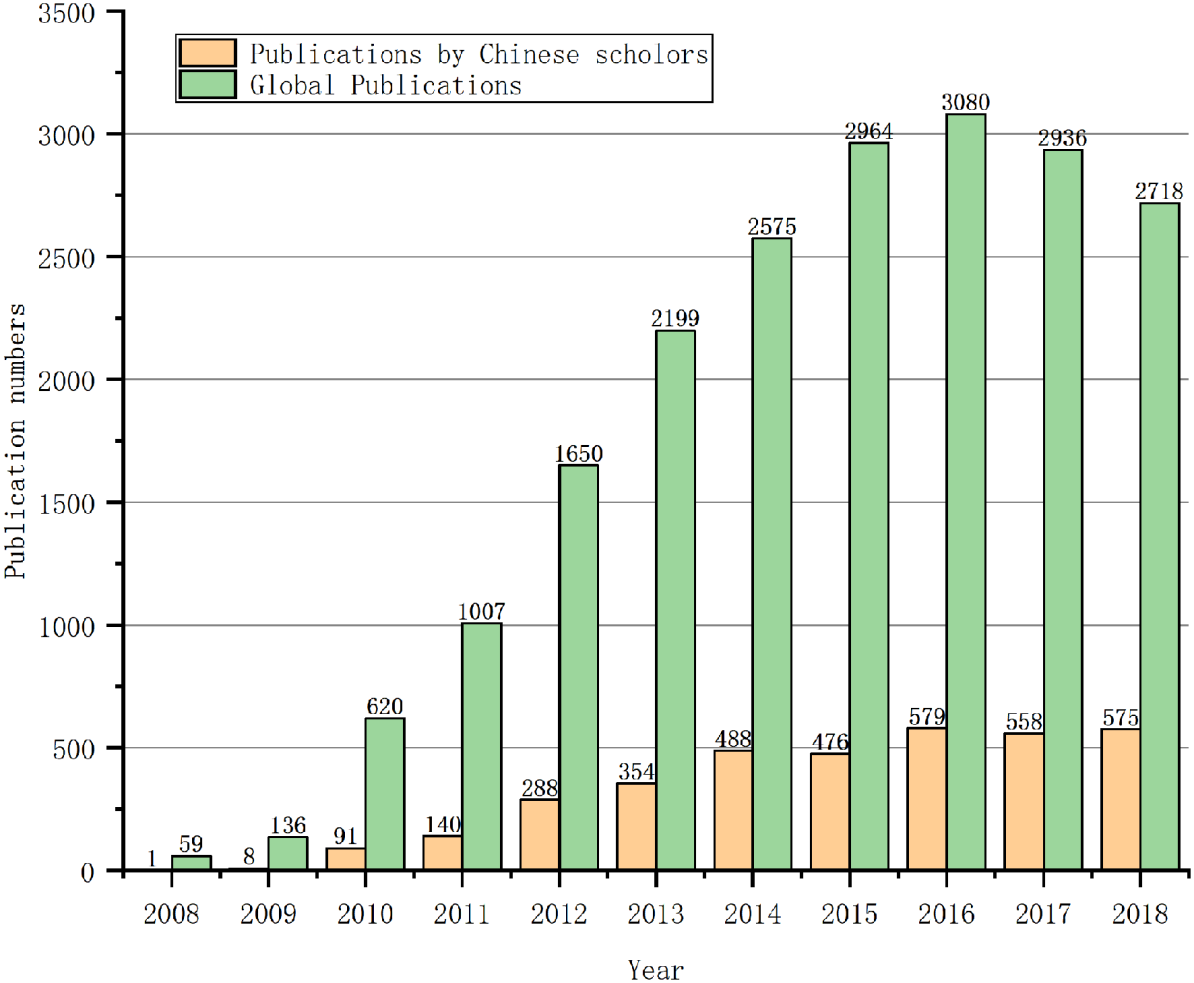
Annual publications by Chinese scholars and worldwide scholars in the field of SG.

Figure 2 presents a comparative analysis of the publication numbers of Chinese and worldwide scholars. Global publication began to decline after peaking in 2016, but the number of publications by Chinese scholars continued to rise until 2018. The number of articles published by Chinese scholars has increased year by year, and more than 570 were published in 2018. Besides, since 2016, Chinese scholars have published more than 550 articles on SG every year.

China's SG publication volume is closely related to policy promotion. For example, in 2010, the National Energy Administration (NEA) started to promote smart grid standardization work by the “*Notice on The Establishment of National Smart Grid Standardization Overall Work Promotion Group*”(National Energy Administration, 2010). In the same year, the State Grid Corporation of China issued the “*Strong Smart Grid Technical Standard System Plan*,” which officially carried out smart grid construction at the national level (Wang, 2010). Since 2010, relevant research began to greatly increase. Similarly, in 2015 the National Development and Reform Commission (NDRC) and NEA issued the “*Guidelines on Promoting the Development of Smart Grid*” (National Development and Reform Commission and National Energy Administration, 2015), which further promoted the development of China's SG. In the following year, China published more than 100 articles on SG. In 2016, NDRC and NEA released “*the 13th Five-Year Plan of Electric Power Development (2016–2020)*” (National Development Reform Commission and National Energy Administration, 2016) which proposed to optimize the power grid structure and further promote the construction of the smart power grid. From 2016 to 2018, the number of papers published in this period was maintained at over 550. In 2020, NDRC and the Ministry of Justice issued “*the Opinions on Accelerating the Establishment of Green Production and Consumption Regulations and Policy System*” (National Energy Administration Ministry of Justice, 2020) which proposed to increase policy support for distributed energy, smart grid, energy storage technology, and multi-energy complementary technology. It is expected that relevant policies will still be introduced in the 14th 5-Year Plan to promote the development of SG in China, and research literature on SG in China will continue to be maintained in the next few years.

Common bibliometric indicators are used in Table 1, for example, TP (total publication) refers to the total number of publications per year, AN (author number) represents the number of authors and can be used to describe the strength of collaboration between authors, TC (total citation) refers to the number of citations, usually used to describe the influence of the literature, RN (reference number) refers to the number of references cited, which is generally used to describe the basis of the literature research, and TC/TP represents the average annual citation amount of the literature which reflects the citation situation of the literature issued by Chinese scholars (Yu et al., 2020).

The number of citations per article by Chinese scholars in the SG field averaged <10 in 2008–2018 and reached the highest level of 22 in 2013. The cited frequency in China is relatively low, among which only two papers were cited more than 1,000 times, one paper was cited more than 500 times, and 1,722 articles have not been cited since publication.

### The Most Productive Chinese Institutions

The top 10 institutions have published a total of 2010 papers, accounting for 56.5%. These 10 institutions are China's main research institutions in the SG field. Table 2 shows some characteristics of these 10 institutions, such as TP, TC, and TC/TP. SGCC (State Grid Corporation of China) ranks first with 644 articles and accounts for 18.1%. Then there are NCEPU (North China Electric Power University), THU (Tsinghua University), CAS (Chinese Academy of Sciences), and SJTU (Shanghai Jiaotong University). There are two institutions whose TP quantity is >300, six institutions between 100 and 200. Both institutions ranked 9 and 10 have a TP quantity <100.

**Table 2:** Most productive Chinese institutions on SG.

**Rank**	**Institution**	**TP**	**TC**	**TC/TP**	**h-index**	**Cited interval^a^**				
						**≥300**	**≥200**	**≥100**	**≥50**	**≥10**
1	SGCC	644	1,652	2.6	17	0	1	1	2	38
2	NCEPU	348	1,653	4.8	19	0	1	1	4	39
3	THU	177	3,810	21.5	28	2	1	2	15	37
4	CAS	152	4,535	29.8	29	3	2	2	8	30
5	SJTU	149	1,298	8.7	20	0	0	2	3	25
6	ZJU	148	3,045	20.6	26	1	2	6	7	32
7	HKU	108	2,409	22.3	26	0	3	5	11	63
8	SEU	103	545	5.3	12	0	0	1	1	12
9	XJTU	95	1,256	13.2	17	0	1	0	5	25
10	HUST	86	1,850	21.5	18	1	0	1	5	19

*TP, total publication; TC, total citation. cited interval refers to the annual total number of TC references interval*,

a *the interval ≥200 in cited interval means 200≦cited interval <300, and all others are similar intervals. Institution abbreviation: SGCC, State Grid Corporation of China; NCEPU, North China Elect Power University; THU, Tsinghua University; CAS, Chinese Academy of Sciences; SJTU, Shanghai Jiaotong University; ZJU, Zhejiang University; HKU, University of Hong Kong; SEU, Southeast University; XJTU, Xi'an Jiao Tong University; HUST, Huazhong University of Science and Technology*.

In terms of TC/TP, the highest is CAS, with an average of 29.8 citations per document. The second to the fifth most cited institutions are HKU (University of Hong Kong) (22.3), THU (21.5), HUST (Huazhong University of Science and Technology) (21.5), and ZJU (Zhejiang University) (16.9). It should be pointed out that SGCC is the institution with the most articles published, but its TC/TP ranked only 10th.

H-index gives an estimate of the importance, significance, and the broad impact of a scientist's cumulative research contributions (Hirsch, 2005). The CAS ranks first in the h-index with the value of 29, followed by THU (28), ZJU, and HKU with an h-index value of 26. There is a big difference between TP and h-index in some institutions, such as HUST which was tenth in TP, which ranks 7th in the h-index, and NCEPU, which ranks 2nd in TP but 6th in h-index.

Table 2 also gives new indicators: cited interval. Cited interval ≥300 refers to the number of literature published by an institution whose citation quantity is ≥300. In this interval, only three papers had been published by the CAS. Besides, one institution had published two articles, two institutions published only one article in this interval, and six institutions did not publish any literature. In institutions with more than 10 citations, HKU (82) ranks first, followed by THU (57), ZJU (48), CAS (45), and NCEPU (45). Cited interval can reflect the academic influence of relevant institutions.

In terms of the frequency of institutional citation, the literature published by the CAS in the SG field was cited the most, more than 4,500 times, with an average of 29.8 citations per article. According to the citation interval, only seven papers were cited more than 300 times in the top 10 institutions, 11 papers were cited more than 200 times, and most of the papers were cited <10 times.

The State Grid Corporation of China (SGCC) published the most literature, but the Chinese Academy of Science (CAS) had the highest h-index, cited interval >300 had more literature than the other institutions, indicating that CAS was the most influential institution in the SG field in China.

### The Most Productive Journals

Overall, 3,558 articles by Chinese scholars were published in 1,268 journals, with an average of 2.8 articles published in each journal. As far as productive journals are concerned, Table 3 shows the top 10 most productive journals published by Chinese scholars on SG and other relevant information. The top 10 journals had published 478 Chinese scholars' papers in the SG field, accounting for only 13.8%. Nevertheless, 41% of journals published only one article and 14% of journals published two articles on the above.

**Table 3:** The top 10 most productive journals on SG by Chinese scholars.

**Rank**	**Journal**	**IF (2018)**	**IF (5 years)**	**TP**	**TC**	**TC/TP**	**Cited interval**				**h**	**The most cited article**	**Times cited**
							**≥200**	**≥100**	**≥50**	**≥20**			
1	IEEE T SMART GRID	10.486	10.607	142	5,795	40.8	4	5	29	44	121	Li et al., 2010	418
2	ENERGIES	2.707	2.99	71	524	7.4	0	0	0	7	64	Yu et al., 2012	39
3	IEEE ACCESS	4.098	4.54	54	571	10.6	0	0	2	7	56	Yu et al., 2017	95
4	IEEE T IND INFORM	7.377	8.423	53	1,893	35.7	2	3	4	13	100	Su et al., 2012	349
5	APPL ENERG	8.426	8.558	34	1,147	33.7	0	2	7	7	162	Wang et al., 2015	126
6	INT J ELEC POWER	4.418	4.262	30	666	22.2	1	0	2	4	100	Tan et al., 2013	261
7	RENEW SUST ENERG REV	10.556	11.239	30	1,020	34.0	0	3	3	9	222	Zhou et al., 2016	156
8	IEEE T POWER SYST	6.807	8.143	29	931	32.1	0	1	5	13	221	Zhong et al., 2013	141
9	IET GENER TRANSM DIS	3.229	3.432	25	232	9.3	0	0	0	3	94	Xiao et al., 2012	28
10	IEEE T IND ELECTRON	7.503	8.459	24	1,168	48.7	0	5	3	7	236	Strasser et al., 2015	164

*IF (impact factor), the data were from the Journal Citation Reports database; TP, total publication; TC, total citation; h, h-index. Abbreviation of the journal name: IEEE T SMART GRID, IEEE Transactions on Smart Grid; IEEE T IND INFORM, IEEE Transactions on Industrial Informatics; APPL ENERG, Applied Energy; INT J ELEC POWER, International Journal Of Electrical Power & Energy Systems; IEEE T POWER SYST, IEEE Transactions On Power Systems; RENEW SUST ENERG REV, Renewable & Sustainable Energy Reviews; IEEE T IND ELECTRON, IEEE Transactions On Industrial Electronics, and IET GENER TRANSM DIS, IET Generation Transmission & Distribution*.

Journal publications are relatively scattered. *IEEE Transactions on Smart Grid*, have published only 142 papers in the past 11 years. Among the top 10 journals, eight have an IF (2008) index >4. The largest IF (2008) is *Renewable & Sustainable Energy Reviews* with a value of 10.556, followed by *IEEE Transactions on Smart Grid* (10.486), and Applied Energy (8.426).

On the other hand, the largest TC/TP journal is *IEEE Transactions on Industrial Electronics*. The average number of citations for Chinese scholars published in this journal is 48.7, followed by *IEEE Transactions on Smart Grid* (40.8), and *IEEE Transactions on Industrial Informatics* (35.7). In terms of h-index, the highest value of *IEEE Transactions on Industrial Electronics* is 236, followed by *Renewable & Sustainable Energy Reviews* (222), and *IEEE Transactions on Power Systems* (221). Table 3 also shows some indicators, such as the most cited literature in the journal and the number of citations, which can help scholars quickly find the important SG literature in the target journal.

Literature in the field of China's SG was mainly distributed in *Engineering, Electrical & Electronic, Computer Science, Energy & Fuels, Telecommunications, Automation & Control Systems*, and *Materials Science*, indicating that there are many interdisciplinary types of research on SG, and that authors of different disciplines are interested in SG.

*IEEE Transactions on Smart Grid* published the highest number of articles by Chinese scholars in 2008–2018, and it is also ranked second in IF and second in TC/TP, indicating that it is the most important journal in the SG field in terms of volume and influence.

### The Most Cited Papers

Since 2008, Chinese scholars have published many influential papers in the SG field. Table 4 lists the top 20 most cited papers in the SG field by Chinese scholars. Among them, the literature published by Pan et al. (2013) has been cited more than 1,650 times, and the other paper that was cited more than 1,000 times is Cheng and Chen (2012), both of which were completed by a single institution. Among the top 20 publications, multi-country cooperation accounted for 75% and multi-institution cooperation 80%. It can be seen that multi-country cooperation and multi-institution cooperation play an important role in high-impact research results in SG.

**Table 4:** The top 20 most cited papers on SG by Chinese scholars.

**Rank**	**Most cited documents**	**TC**	**Citation/year**	**AN**	**RN**	**IN**	**CN**
1	Pan et al., 2013	1,657	237	3	197	1	1
2	Cheng and Chen, 2012	1,300	163	2	217	1	1
3	Chen et al., 2014	852	142	3	155	3	2
4	Sun et al., 2013	475	68	11	56	4	2
5	Hu et al., 2013	437	63	3	278	1	1
6	Li et al., 2010	417	42	8	52	8	3
7	Wang et al., 2013	410	59	10	59	2	2
8	Su et al., 2012	349	44	4	91	2	2
9	Lin et al., 2017	290	97	6	163	5	2
10	Zhong et al., 2014	287	48	4	29	3	2
11	Deng et al., 2015	267	54	4	95	2	2
12	Rahimi-Eichi et al., 2013	262	38	4	45	3	2
13	Varaiya et al., 2011	261	29	3	44	3	3
14	Tan et al., 2013	260	38	3	52	2	1
15	Tsui and Chan, 2012	246	31	2	19	1	1
16	Zhang et al., 2012	243	31	6	10	2	2
17	Gao et al., 2012	237	30	5	203	4	2
18	Zhao et al., 2015	219	44	4	592	2	2
19	Wu et al., 2012	219	28	3	38	3	2
20	Liu et al., 2012	217	28	5	116	5	2

*TC, total citation; AN, author number; RN, references number; IN, institution number; CN. country number*.

Table 4 also shows the cited references with high citation frequency in the SG field in China. Through the cited references with high citation frequency, research hotspots similar to the co-occurrence of keywords can be found. The top 20 cited literature can be divided into the following research topics: smart grid (rank 6, 10, 13), batteries and energy storage (rank 1, 2, 4, 5, 7, 12, 14, 19), EV (rank 8, 18), IoT (rank 9), demand response (rank 11, 15), smart grid communication technology (rank 16, 17, 20), and big data technology (rank 3).

The overall adoption of SG and dispatching synchronizer research are the main topics focusing on SG's impact on traditional power grids. For example, Li et al. divided SG into smart control center, smart transmission grid, and smart substation, and regarded it as an integrated system to discuss its characteristics and performance (Li et al., 2010). Zhong et al. focused on the self-synchronized synchronverters of the smart grid (Zhong et al., 2014). Varaiya et al. have designed a risk-and-speed dispatch mode to improve grid efficiency (Varaiya et al., 2011).

Batteries and energy storage account for the highest proportion of literature citations, which on the one hand reflects emphasis on energy storage in the SG field in China. Among them, three articles discussed room-temperature sodium-ion batteries (Pan et al., 2013; Sun et al., 2013; Wang et al., 2013), metal-air batteries were mentioned in one article (Cheng and Chen, 2012), LI4TI5O12-based electrodes for lithium-ion batteries were discussed in one article (Zhao et al., 2015), and one paper wrote about high-voltage lithium-ion batteries (Hu et al., 2013). In addition to the research of battery technology, the battery management system plays an important role in improving battery performance (Rahimi-Eichi et al., 2013). Energy storage systems (ESSS) realize comprehensive battery management from a wider range to improve the efficiency of battery use (Tan et al., 2013). EV are both users and suppliers in the smart grid, and their energy storage role is well-recognized. Wu et al. programmed a new game-theoretic model to understand the interactions among EV and aggregators in a vehicle-to grid (V2G) market (Wu et al., 2012). Su et al. focused on transportation electrification and introduced the current situation and prospect of electric vehicles in the field of industrial information systems (Su et al., 2012).

Network communication technology is the basis of smart grid operation, mainly including network security and privacy issues (Liu et al., 2012). The Machine-to-machine (M2M) Communications Paradigm (Zhang et al., 2012) and systematic Review of Communication/Networking Technologies in Smart Grid (Gao et al., 2012) discussed this.

IoT and big data play an irreplaceable role in the smart grid, Lin et al. reviewed the IoT in smart grid technology (Lin et al., 2017), similarly, Chen et al. reviewed the background and state-of-the-art of big data (Chen et al., 2014), These reviews play an important role in advancing the IoT and big data applications in SG.

Another important theme in China's SG field is demand response, which promotes load forecasting and management in smart grids (Tsui and Chan, 2012), power grid dispatch, and the electric market (Deng et al., 2015). The theme of highly cited literature in the SG field in China is similar to that of the co-occurrence of keywords, which can further reflect the research hotspots in the SG field in China.

## Visualization Analysis

### Co-authorship Analysis

Co-authorship analysis has been widely used in the cooperative research of scientific research institutions and researchers. Although it is somewhat similar to the citation network (Garfield, 1979), co-authorship is a temporal and collegial relationship, which is fairer than the anonymity of citation (Liu et al., 2005).

#### Co-authorship Between Countries

**Figure 3:**
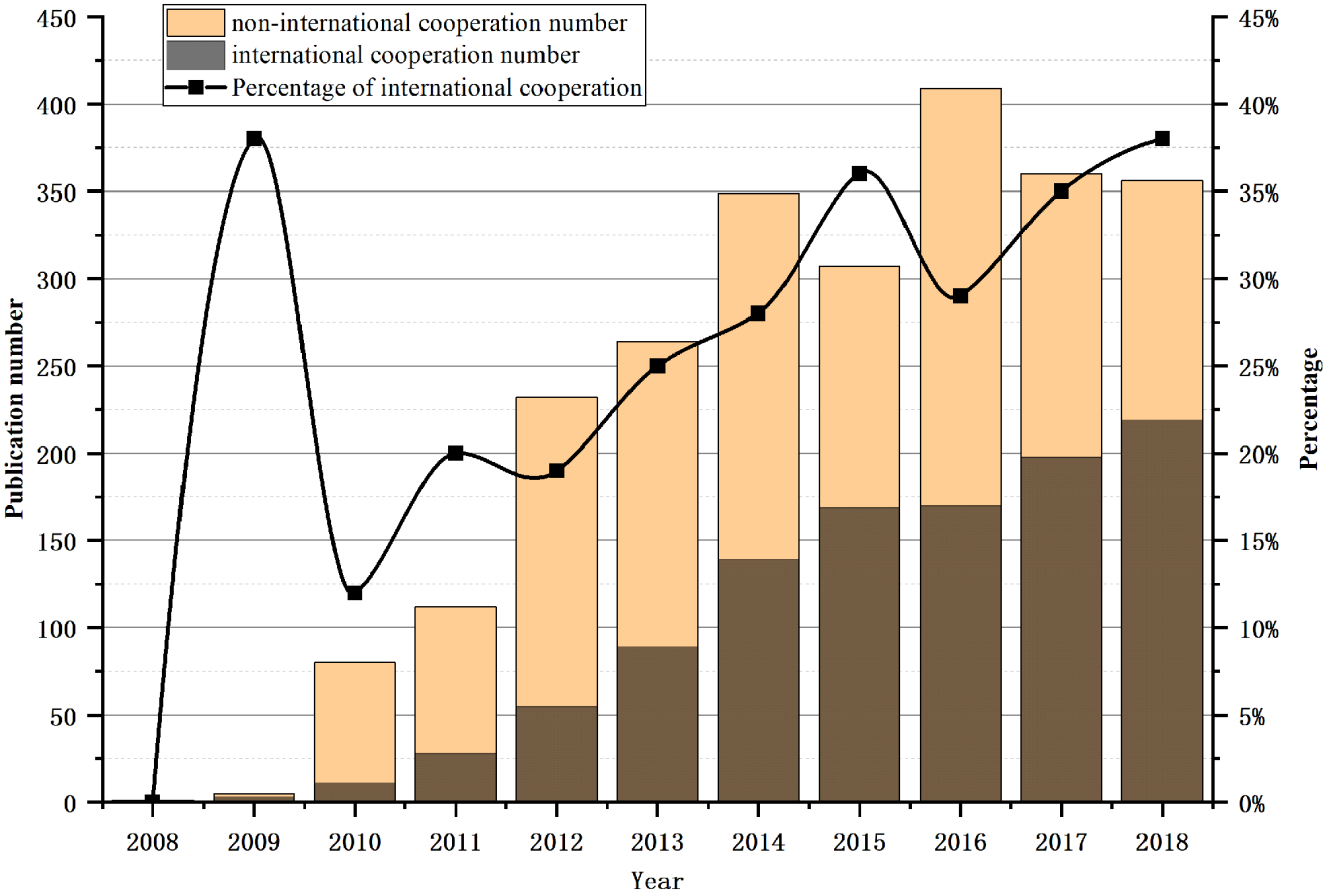
Annual literature distribution of international and non-international cooperation.

Figure 3 shows the publication numbers of international cooperation and non-international cooperation, as well as the percentage of international collaborative publications. The number of international cooperation and non-international cooperation papers all showed a trend of growth and a larger percentage of international cooperation in 2009. This growth could possible be due to more published learning technology and experience from abroad at the initial stage of China's SG research. After 2010, the percentage of international cooperation fluctuated but has since been on the rise, which shows that the field is increasingly attracting the attention of more institutions.

**Figure 4:**
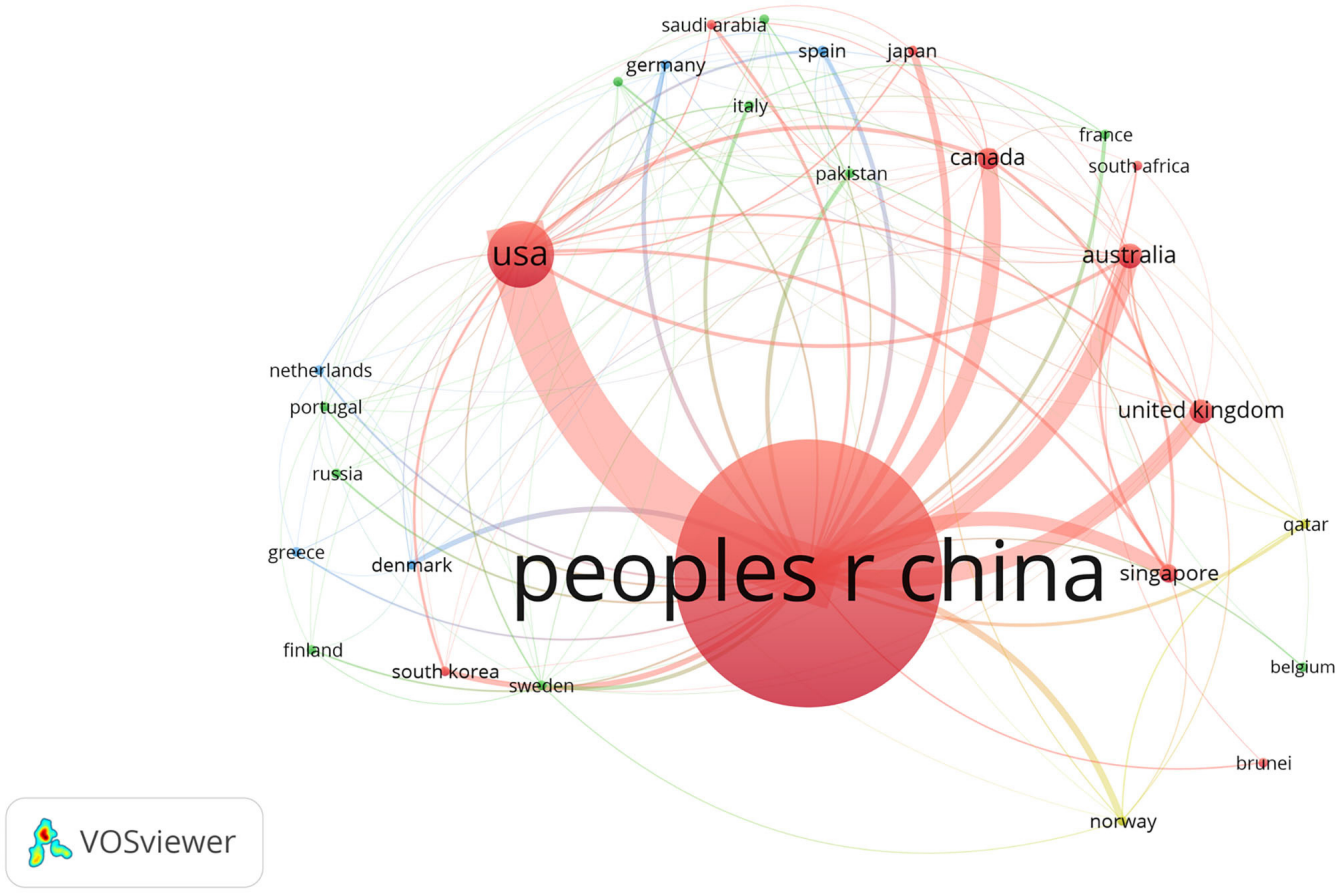
Country co-authorship network in the field of the SG field. The larger the node is, the more papers published in cooperation between the country and China, and the thicker the connection between the nodes, the closer the cooperation between the two countries is. VOSviewer parameters: counting method: full counting, minimum occurrences 5, a total of 28 countries, normalization using LinLog/modularity method, layout parameters: attraction = 6, repulsion = 0.

The rate of international cooperation in the SG field is on the rise, but which countries have closer cooperation with China? Figure 4 shows the network of countries with international cooperation with China. The United States is the country with the most cooperation, with 508 cooperative articles. Followed by 130 articles from Australia, 117 articles from the United Kingdom, and 109 articles from Canada. The larger the node is, the more papers published in cooperation between the country and China are, and the thicker the connection between the nodes, the closer the cooperation between the two countries is.

Figures 3, 4 show China's cooperation with other countries in the SG field. The current research also indicates that cooperation with foreign countries in the SG field is increasing. The average cooperation rate from 2008 to 2018 was 25.5%, and the cooperation rate reached 38% in 2018. The United States and China have the largest number of collaborative literature. Other countries that continue to work closely with China are Australia, the United Kingdom, and Canada. The overall increase of foreign cooperation rate indicates that Chinese scholars are paying more attention to cooperation with foreign countries in the SG field.

#### Co-authorship Between Institutions

**Figure 5:**
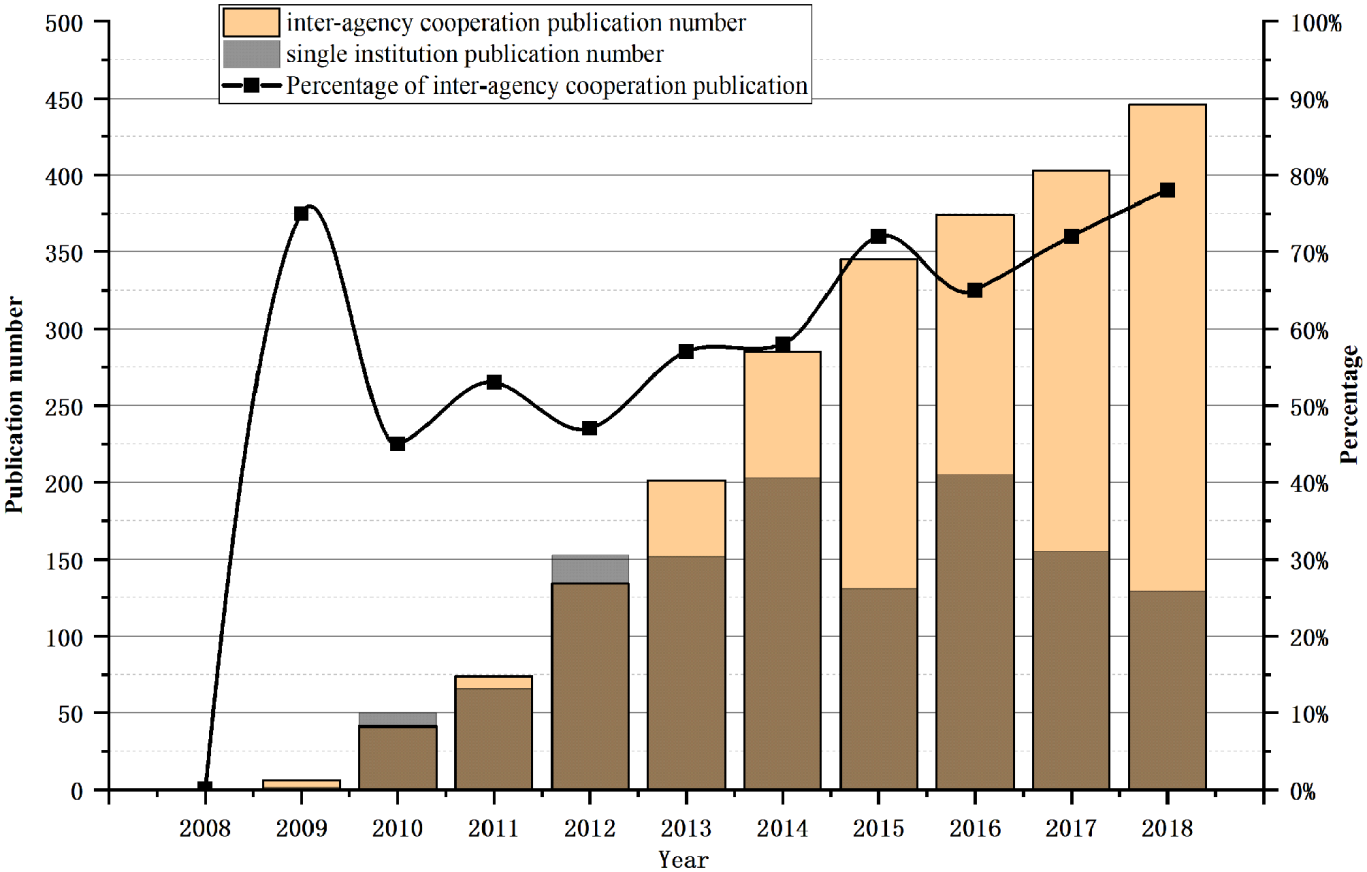
Annual literature distribution of inter-agency and single institution.

As indicated in Figure 5, a total of 1,052 institutions have published literature in the SG field. The proportion of papers for inter-agency collaboration increased substantially in 2009, similar to the increase in country collaboration in 2009. In 2013, the number of inter-agency collaborations began to increase significantly, and the number of inter-agency collaboration papers in the following years showed an increasing trend. In terms of percentage, inter-agency collaboration was at 72% in 2015, fell to 65% in 2016, and then kept on rising, accounting for 78% in 2018.

**Figure 6:**
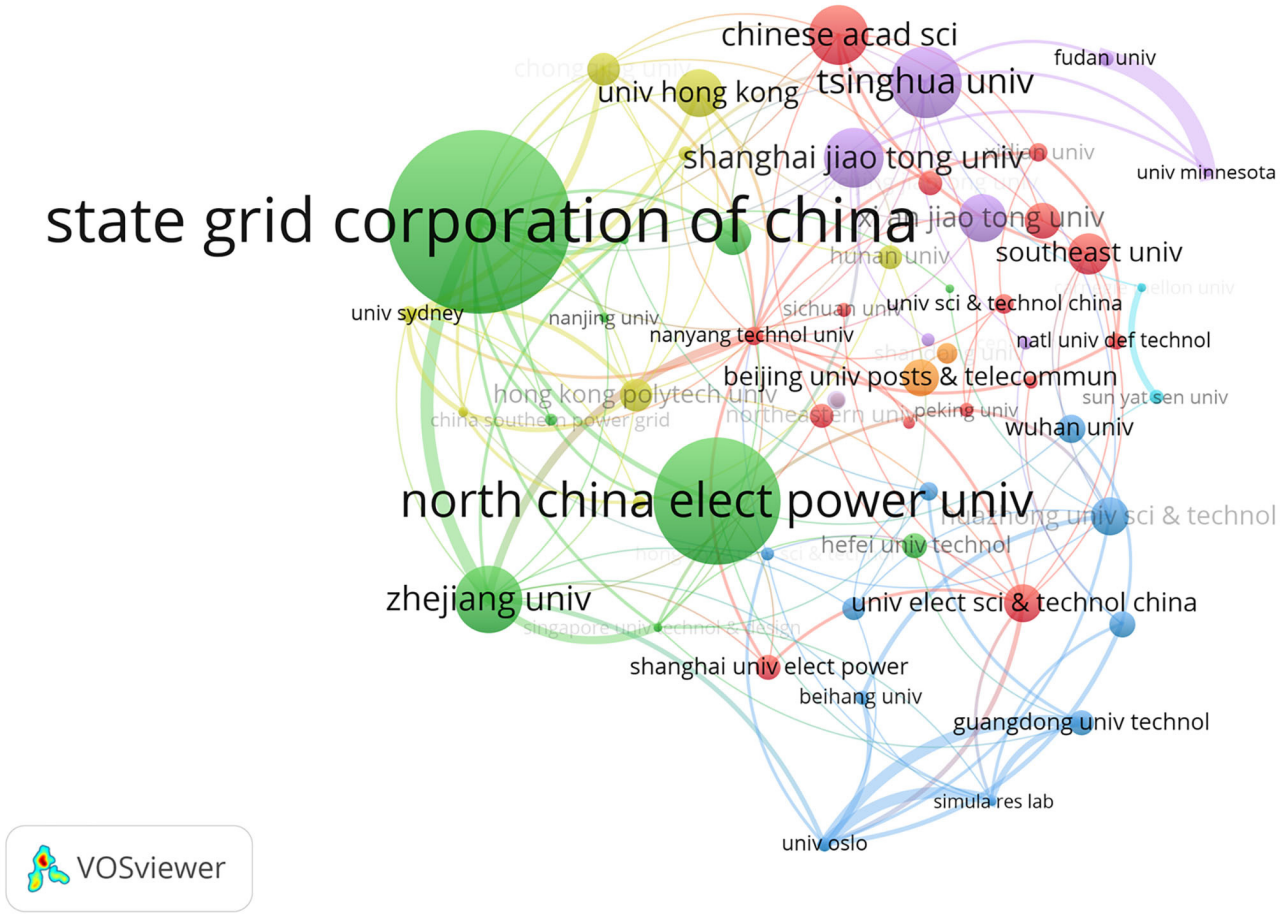
Institution co-authorship network in China in the field of SG. VOSviewer parameters: counting method: full counting, minimum occurrences 10, a total of 61 institutions, normalization using LinLog/modularity method, layout parameters: attraction = 8, repulsion = 0.

To figure out the institutional cooperation in the SG field research by Chinese scholars, VOSviewer was used to make the institutional cooperation network as shown in Figure 6. The size of the circle in Figure 6 is equal to the number of posts issued by the organization, and the thickness of the line represents the cooperative relationship between the organizations. For example, Fudan University and the University of Minnesota have the closest cooperation relationship (11 times), and then the State Grid Corporation of China and Zhejiang University (8 times), Guangdong University Technology, and University of Oslo (7 times).

Next we consider the cooperation clustering. The different colors in Figure 6 represent the clustering of the VOSviewer to the institution, thereby revealing the collaboration between the institutions. Green is the cluster with the largest volume of publications, mainly including 10 institutions such as the State Grid Corporation of China, Zhejiang University, North China Electric Power University, and Tianjin University. The second is the purple clustering, which mainly represents Tsinghua University, Shanghai Jiao Tong University, Xi'an Jiao Tong University, Fudan University, and the University of Minnesota. Red clustering is far away, major representative institutions include the Chinese Academy of Science, Southeast University, Beijing Jiao Tong University, and the University of Electric Sci & Technology China. Blue clustering includes Huazhong University of SCI & Technology, Wuhan University, and the University of Oslo.

### Co-occurrence Analysis

Co-occurrence analysis has been widely used in bibliometric analysis, the method of co-word analysis has been put forward since the 1980s (Callon et al., 1983). Keywords co-occurrence has been used at the forefront of the analysis of hot topics (Chen, 2017).

#### Author-Keywords Co-occurrence of Five Most Productive Institutions

**Figure 7:**
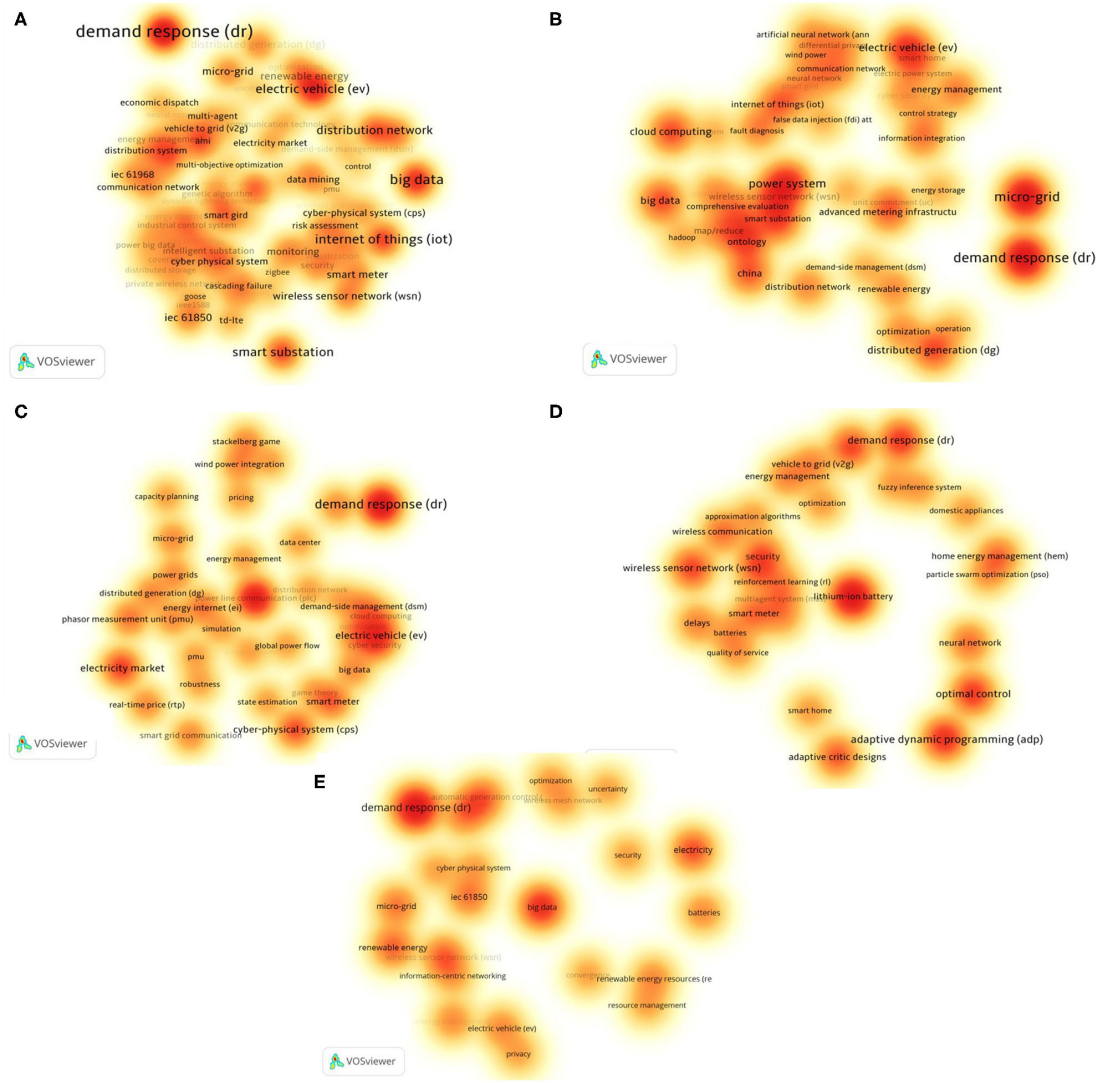
Main research topics in the top five most productive institutions. Author-keywords are selected, counting method: full counting, search keywords such as smartgrid, smart grid, and smart grids are not included. The brighter the color, the more frequently the keyword appears. VOSviewer parameters: normalization method: LinLog/modularity, attraction = 8, repulsion = 1, clustering: resolution = 1, min. The cluster size: 1, density kernel width: item density = 1.18. **(A)** The research topic of the State Grid Corporation of China. A total of 1,802 keywords, minimum occurrence is 3, 131 keywords were obtained. **(B)** The research topic of North China Elect Power University. A total of 1,045 keywords, minimum occurrence is 3, 49 keywords were obtained. **(C)** The research topic of Tsinghua University. A total of 581 keywords, minimum occurrence is 3, 40 keywords were obtained. **(D)** The research topic of the Chinese Academy of Sciences. A total of 490 keywords, minimum occurrence is 3, 31 keywords were obtained. **(E)** The research topic of Shanghai Jiaotong University. A total of 613 keywords, minimum occurrence is 3, 26 keywords were obtained.

Different institutions have different research emphases. Figure 7 shows the keyword co-occurrence map of the five institutions with the most articles published in the SG field in China, which can be utilized to understand the research emphases of these five institutions. The hotspots of each institution are displayed as the depth of color in Figures 7A–E. The brighter the color, the more frequently the keyword appears. Demand response is the field with the brightest color in Figures 7A–E, which indicates that each institution regards the demand response as the research hotspot. The most frequent occurrence is State Grid Corporation of China (SGCC) (38 times), while the other institutions use this keyword more than 15 times. EV appeared in research hotspots of the State Grid Corporation of China (SGCC), North China Electric Power University (NCEPU), Tsinghua University (THU), and Shanghai Jiaotong University (SJTU). Big data is a common research hotspot of NCEPU, SGCC, and SJTU, while micro-grid is a research hotspot of NCEPU and SJTU.

In addition to the common research hotspot of demand response, different institutions have different research hotspots. For example, SGCC's research mainly focuses on big data, IoT, EV, smart substation, distribution network, cloud computing, WSN, distributed generation, micro-grid, renewable energy, IEC61850, and smart meter. SGCC, as China's largest network service provider, is committed to building a strong smart grid based on Ultra-High Voltage (UHV). This was proposed in 2009 by the smart grid development framework: one goal, two main lines, three stages, and four systems, five connotations, and six sections (Yu et al., 2012) including different aspects of smart grid construction. SGCC focused on IoT by smart electricity meters to improve data collection and analysis capabilities, the effectiveness of energy efficiency can be realized through big data and distributed systems. To improve energy efficiency, the use of electric vehicles and products such as vehicle-to-grid (V2G) that may affect the load on the grid needs to be considered. SGCC is the backbone of China's smart grid construction.

Except for SGCC, the other top four publishers are all scientific research institutions or universities, and their research hotspots are related to the characteristics of the institutions. For example, the hotspots of NCEPU (North China Electric Power University) involve various aspects of the power system, such as distributed generation, micro-grid, IoT, cloud computing, big data, and energy management information systems such as EV and energy storage. As a university focusing on power research, its University Council includes SGCC and other national key power companies and the China Electricity Council. The power system has always been its research topic. Its research hotspot includes SG technology foundation, such as IoT, power WSN, and many aspects of the power system.

Research hotspots for Tsinghua University mainly include EV, electricity market, cyber-physical systems (CPS), and smart meters. Among them, CPS is its unique research hotspot. Under the academic advantages of Tsinghua University, CPS achieves breakthroughs in remote control of distribution networks and reliable, safe, and efficient transmission and distribution of energy. CAS research mainly focuses on adaptive dynamic programming, optimal control, micro-grid, WSN, adaptive critic designs, security, neural network, and home energy management. CAS also pays attention to the research of power system planning and other related policies and algorithms. SJTU mainly studies renewable energy, WSN, micro-grid, IEC61850, batteries, energy management, and EV. SJTU pays more attention to renewable energy utilization, WSN, micro-grid, and IEC61850.

#### Author-Keywords Co-occurrence Analysis With Three Stages

Based on author-keywords co-occurrence research, from 2008 to 2018, a total of 7,886 author-keywords were used. A total of 6,308 author-keywords appeared only once, and 123 author-keywords had a frequency >10.

**Figure 8:**
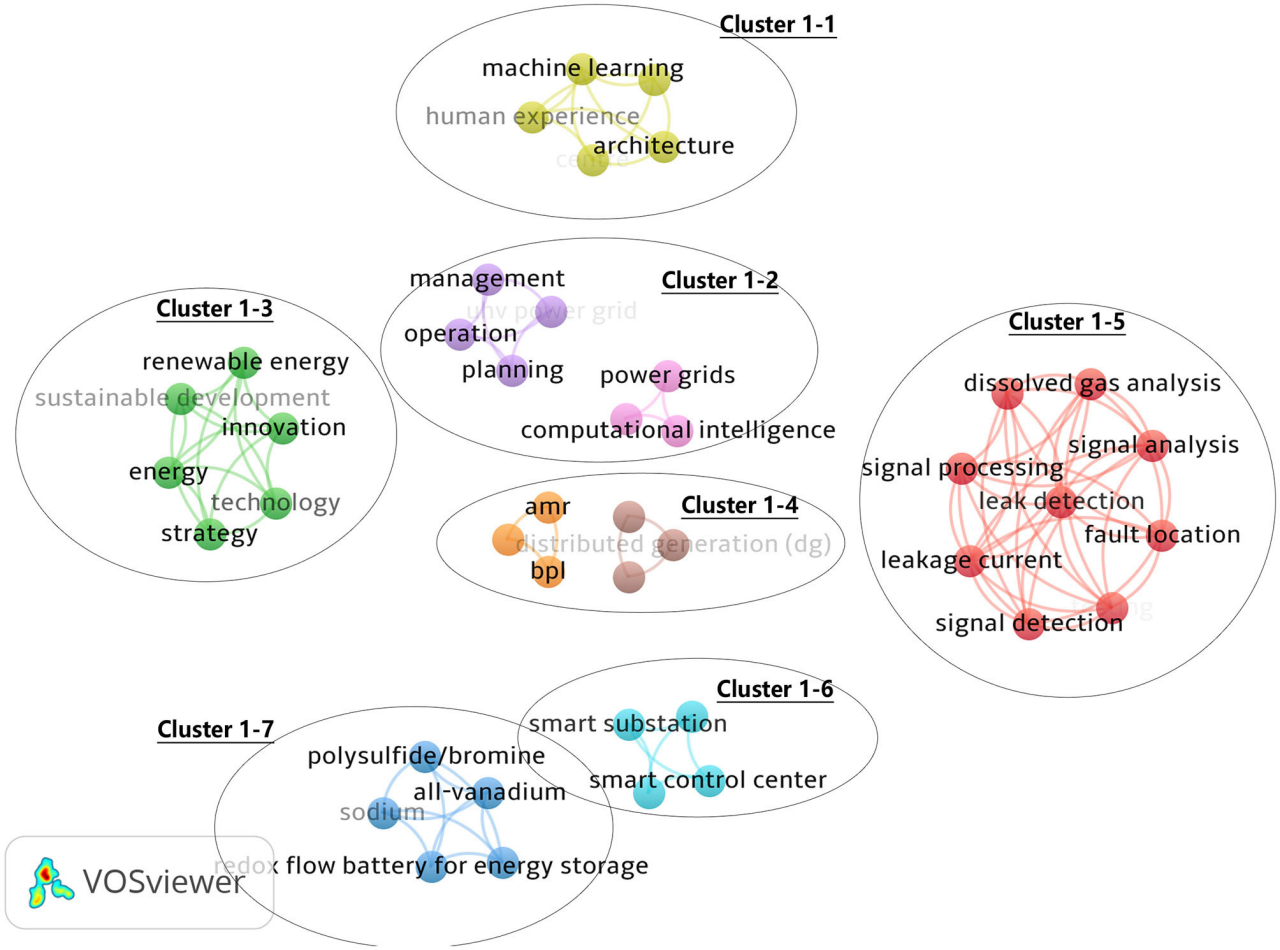
Author-keyword co-occurrence network of SG publications in China, 2008–2009. A total of 42 keywords, VOSviewer parameters: minimum occurrence is 1, normalization method: LinLog/modularity, attraction = 3, repulsion = 0, clustering: resolution = 1 min. The cluster size: 1. Search keywords such as smartgrid, smart grid, and smart grids are not included.

**Figure 9:**
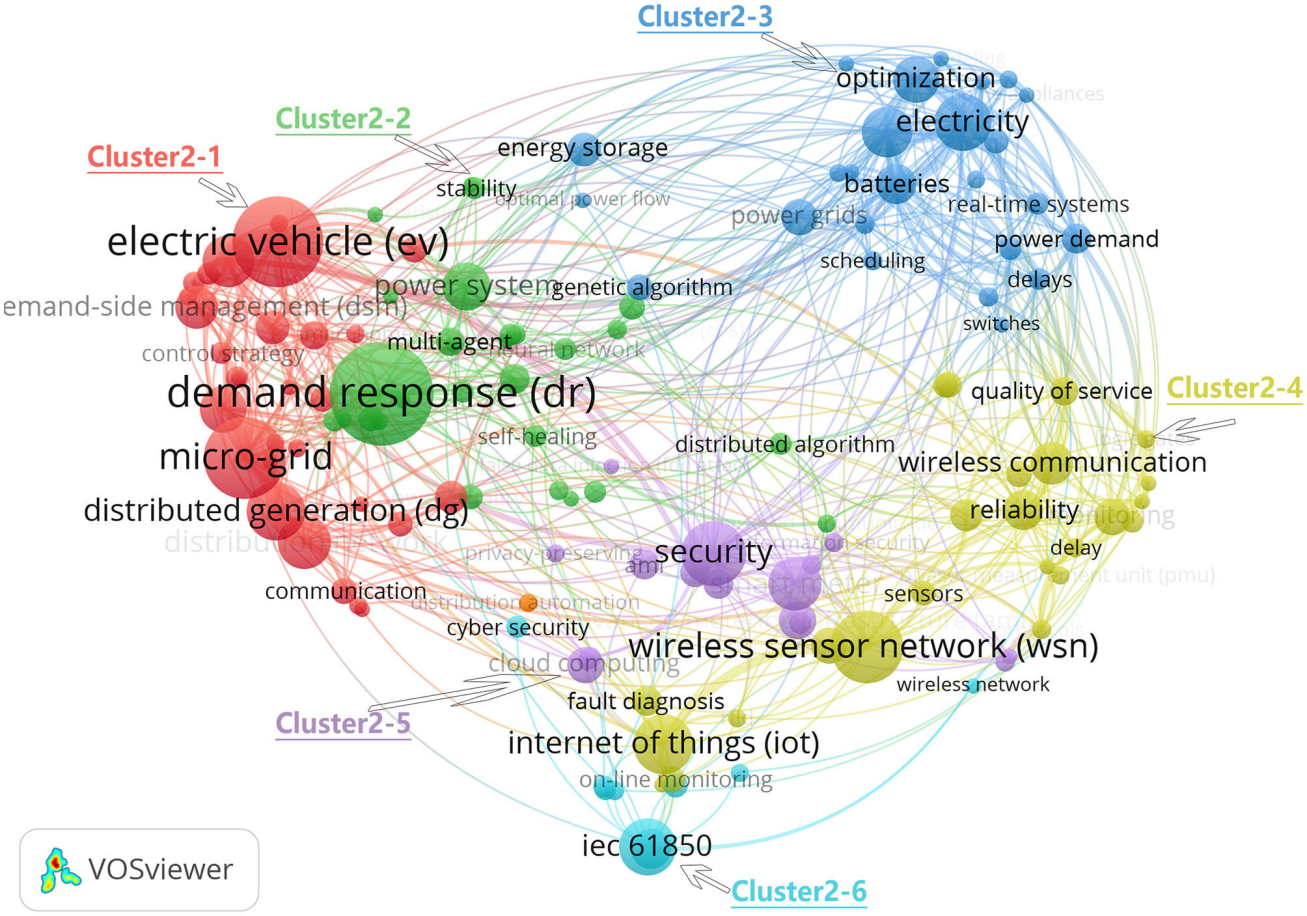
Author-keywords co-occurrence network of SG publications in China, 2010–2014. A total of 3,185 keywords, VOSviewer parameters: minimum occurrence is 5, normalization method: LinLog/modularity, attraction = 1, repulsion = 0, clustering: resolution = 1, min. The cluster size: 5. Search keywords such as smartgrid, smart grid, and smart grids are not included.

**Figure 10:**
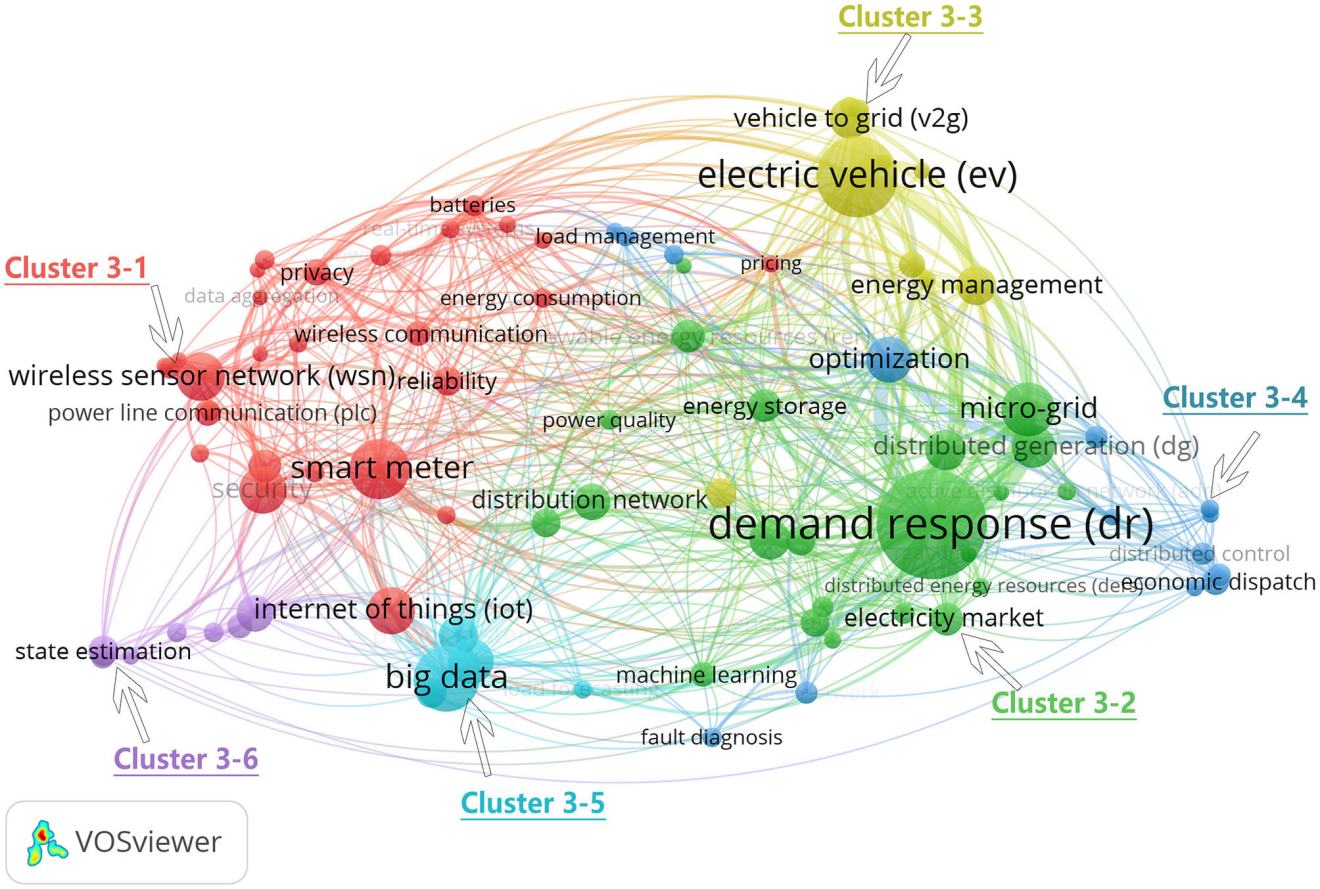
Author-keyword co-occurrence network of SG publications in China, 2015–2018. A total of 5,457 keywords, VOSviewer parameters: minimum occurrence is 10, normalization method: LinLog/modularity, attraction = 1, repulsion = 0, clustering: resolution = 1 min. The cluster size: 1. Search keywords such as smartgrid, smart grid, and smart grids are not included.

In section Annual Publications and Growth Trend, we divided 2008–2018 into three stages. In this section, we will examine the co-occurrence of keywords in these three stages to find the research hotspots in each stage. Figures 8–**10** show the analysis map of keyword co-occurrence in the three stages, in which the node size is equal to the number of co-occurrences of keywords, and the larger the node, the more co-occurrences. The line between nodes represents the number of simultaneous occurrences of two keywords, and the thicker the line is, the more simultaneous occurrences of keywords were present.

Figure 8 is the keywords co-occurrence map of Chinese scholars' publications in the field of SG from 2008 to 2009, with keyword minimum occurrences as 1, 42 keywords were obtained. This diagram is composed of several independent parts, labeled as cluster 1–1 to cluster 1–7. Cluster 1–1 contains the keywords: machine learning, architecture, and human experience, which can be regarded as the application of artificial intelligence in the power system. Cluster 1–2, whose theme is grid planning and management, mainly contains keywords such as planning and management power grids. Cluster 1–3 focuses on renewable energy and sustainable development, including innovation, technology, and strategies. Cluster 1–4 focuses on integrating distributed energy to improve energy efficiency, mainly including keywords such as distributed generation and energy efficiency. Cluster 1–5 is the largest, and its theme is mainly based on artificial intelligence and the automatic fault identification of sensors, mainly including signal analysis, leak detection, signal detection, and other keywords. Cluster 1–6 contains only four keywords: smart substation, vision, smart control center, and smart transmission grid, which can be thought of as the clustering theme for intelligent control of power transmission and distribution. The last cluster 1–7 is mainly about the research of batteries. At this stage, China's SG has just started, and some attempts have been made at the technical level, but the correlation between different topics is weak.

SG in China is at an embryonic stage and is committed to solving the problem of renewable energy consumption. The research mainly focuses on the power grid itself, such as power grid dispatching, intelligent power transformation, and power grid planning. Meanwhile, battery, big data, and machine learning are proposed.

Figure 9 shows the keyword co-occurrence map of Chinese scholars' publications in the SG field from 2010 to 2014. Keywords at this stage are considered more closely related, indicating that the research in the SG field at this stage is more scattered and involves more fields. The top 10 keywords at this stage are demand response, EV, micro-grid, WSN, security, IoT, distribution generation, electricity, IEC61850, and distribution network. VOSviewer automatic clustering produces six clusters, labeled as cluster 2–1 to cluster 2–6. The largest cluster is cluster 2–1, which contains 29 keywords on the integration of distributed energy, including the following keywords: EV, micro-grid, distributed generation, and distribution network. The second-ranking cluster is cluster 2–2, which contains 28 keywords. The theme is DSM to improve energy efficiency. The main keywords include demand response, self-healing, and multi-agent. The next cluster is cluster 2–3, with 25 keywords. The theme is energy storage, batteries, real-time systems, scheduling, and delays. Cluster 2–4 is the technology base of SG, including IoT, WSN, sensors, reliability, quality of service, and other keywords. Security is becoming increasingly important in SG. Cluster 2–5 contains related topics and consists of 16 keywords, including intelligent electricity meter, cloud computing, and advanced metering infrastructure (AMI). The last cluster 2–6 focuses more on the establishment of SG standards, including smart grid standards such as IEC61850.

This stage belongs to the development period of SG in China. Driven by policies and technological progress, research keywords are exploding, which mainly reflect three research themes. Firstly, the SG technology foundation, such as IoT and WSN, are emerging in a concentrated manner, among which smart meters are the most represented in the IoT field. At the same time, studies on SG standards also began to appear, such as IEC61850 related research. Secondly, SG lead to system reconstruction of the power system, traditional power grid power generation, transmission, substation, power distribution, and utilization and scheduling boundaries become blurred, distributed energy, and micro-grid become relatively independent of the interconnected power system, EVs became the new way of energy storage, demand-side management combined with smart meters, have the potential to further enhance energy efficiency. SG has transformed the power system from a traditional independent link into an interconnected and mutually reinforcing internet.

Figure 10 shows the map of keyword co-occurrence of Chinese scholars in the SG field from 2015 to 2018. The minimum total number of occurrences was 5, and 139 keywords were obtained. In the third stage, the research mainly focuses on demand response, EV, big data, smart meter, micro-grid, WSN, security, IoT, optimization, cloud computing, and distributed generation. Similar to the previous stage, the keyword co-occurrence map in this stage is automatically clustered into 6 clusters, labeled as cluster 3–1 to cluster 3–6. The largest cluster is cluster 3–1, whose theme is similar to cluster 2–4. It is the technical basis of SG and includes 28 members, including WSN, IoT, smart meters, power line communication, and reliability. Cluster 3–2 covers all aspects of the power system, such as demand response, energy storage, distributed network, power market, and micro-grid. Its theme is SG's impact on the power system. At this stage, EV becomes an independent cluster (cluster 3–3), indicating that EV are getting more attention because they are both an energy consumer and an energy provider, which is of great significance for energy management. Cluster 3–4 has the theme of power system optimization and contains 14 members. The main keywords are optimization, distributed control, economic dispatch, and load management. Cluster 3–5 is for big data processing; SG generates massive data, which need to be processed by relevant big data methods, including big data, load forecasting, and other keywords. Cluster 3–6 includes cyber-physical systems (CPS), smart substation, cyber-attack, cascading failure, state estimation, cyber security, and false data injection attack, the main theme for the physical information system and its safety.

The third stage is the stability period of China's SG development. Keywords in this stage are more detailed, as shown in Figure 10, the number of nodes with larger diameters have increased significantly, which can be roughly divided into three themes. The first is still the technical basis of SG. In this stage, in addition to the main keywords of the previous stage, such as IoT, WSN, and smart meter, some new features have emerged. If big data become a research hotspot, with the development of SG and more data collection, the problem of data analysis will be raised. At the same time, security has become another important topic. Sensor, data communication, system vulnerability, and other security problems are increasing in SG. Security and stability are the basic requirements of the power system. The second theme is the impact of SG on the power system. Keywords also appear in the trend of decentralization, such as renewable energy, energy management, and energy efficiency. China is facing the grim situation of greenhouse gas emissions. At the same time, the energy internet appears in this stage, which is the depth and development of SG. At this stage, research on EV and big data become independent clusters, and their research becomes more detailed.

Research in China's SG field has obvious interdisciplinary features. For example, the wireless transmission network is an important way to ensure data transmission. It belongs to the application of data communication in the smart power grid. The IoT is an important carrier for smart devices to play their roles. It involves electronics, communication, and other fields. The power system is increasing the proportion of renewable energy, SG can partly solve the intermittent issues. Energy storage is an important method. How to plan as a whole and consider the power system under the influence of smart grid optimization is a problem that academic circles have been exploring, so in the future smart grid, the influence of traditional power systems will be bigger; China has the most complex power grid in the world, with complex power consumption and users. Demand response has always been a hot research topic. Chinese researchers have been seeking to realize optimal dispatching of the power grid through demand response, to guarantee the security and stability of the power grid.

#### The Alluvial Diagram of Author-Keywords

According to the clustering of keywords at different stages in Figures 8–10, the research hotspots at different stages can be roughly seen, but the development and evolution of research hotspots cannot be shown. Therefore, the alluvial diagram is used in this paper to show the evolution of hotspots at different stages.

**Figure 11:**
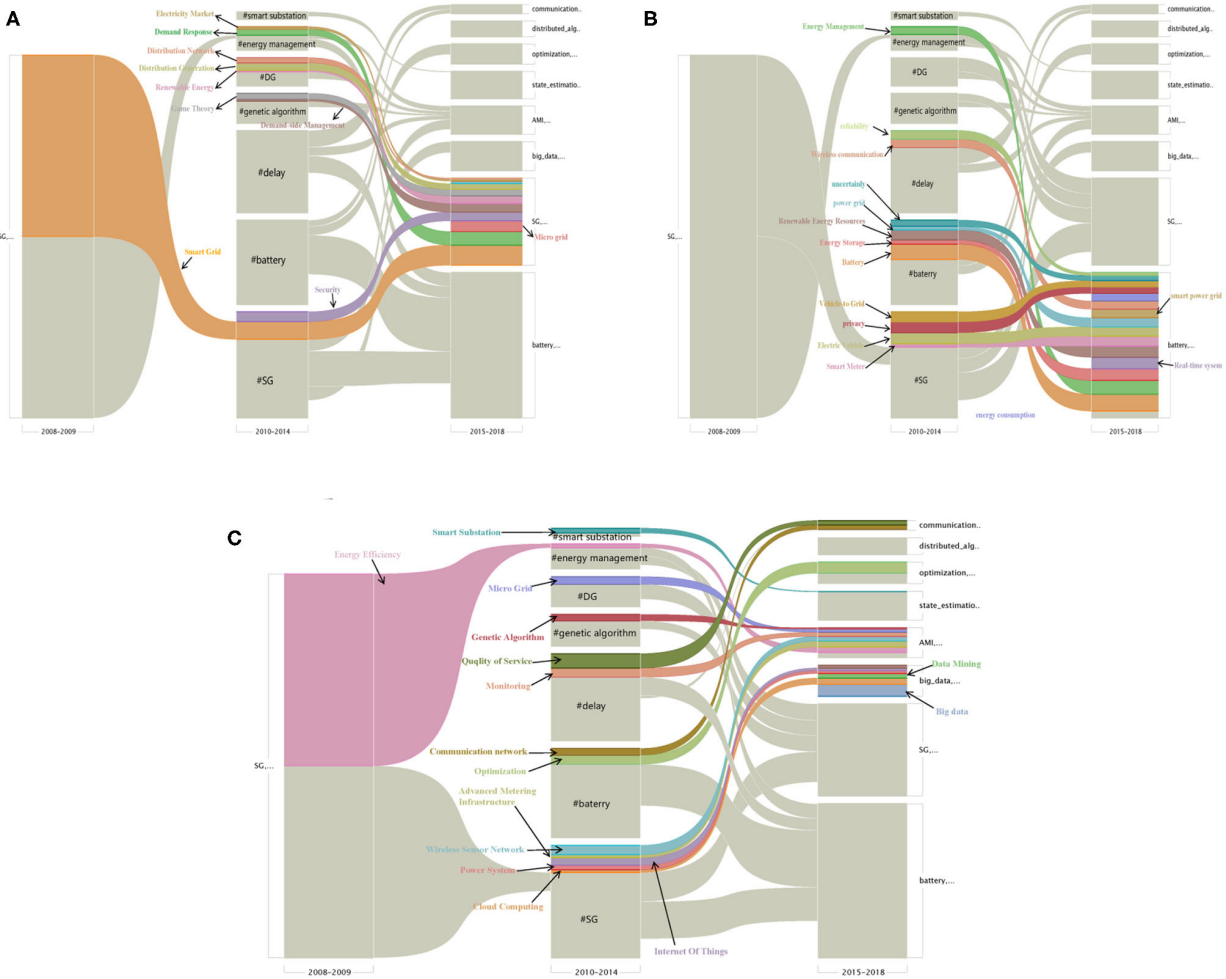
The alluvial diagram of author-keywords in three stages. Significance clustering in 2008–2009, 2010–2014, and 2015–2018 takes up a column in the figure. Each block in a column represents a cluster, and the height of the block reflects the keywords flow for that cluster. Darker color was used to indicate the significant subset of each cluster. All keywords that are clustered in the subset of others in 2015–2018 are colored to highlight the fusion and formation of clusters. **(A)** Flow of keywords in SG clustering. **(B)** Flow of keywords in battery clustering. **(C)** Flow of keywords in other clustering.

Looking for changes in the scientific structure is important for understanding the development of science. Alluvial diagrams are such a tool that reveal stories in the network data and allow us to connect structural and functional changes (Rosvall and Bergstrom, 2010). This article utilizes MapEquation's alluvial diagrams to understand the changing trends of research hotspots in the SG field. Firstly, the data set was pre-processed in CiteSpace (Chen, 2013). It was divided into three stages, 2008–2009, 2010–2014, and 2015–2018, respectively. Three.net files were generated and used to import MapEquation's alluvial diagrams APP. Alluvial diagrams illustrated in Figures 11A–C were finally generated after computing clusters to simplify the network (Rosvall and Bergstrom, 2008)^1^.

In Figure 11, every equivalently colored block in the alluvial diagram presents a cluster in the networks. The change of the cluster structure from one time period to the next period was represented by the merge and divergence in the strips connecting the time 1 and time 2 blocks (Rosvall and Bergstrom, 2010). For example, Figure 11A shows how SG in China evolved from a relatively independent topic to a cluster of many research topics between 2008 and 2018.

The height of the block reflects the size of the cluster, the clusters are ordered from bottom to top by size, with mutually non-significant clusters placed together and separated by a third of the standard spacing. Here we will prefix # with the cluster name to differentiate it with the subset. For example, in the 2015–2018 column in Figure 11A, the cluster #battery at the bottom is the largest, so it has the highest block. Cluster #SG is the second largest, ranking on top of the battery, with smaller blocks. The height of a stream field, going from the subset of a cluster in one column to the subset of a cluster in the adjacent column, which represents the total size of the nodes that make this particular transition. By following all stream fields from a cluster to an adjacent column, it is possible to study in detail the mergers with other clusters and the significant transitions. The block representing cluster #SG is composed of many streams, the largest of which is the orange stream at the bottom, which represents the highest proportion of PageRank for this node (Lambiotte and Rosvall, 2012). The orange stream flows from 2008 to 2009 through 2010–2014 and finally flows into 2015–2018, indicating that this research topic runs through three stages and has been a hot research topic. Similarly, Figure 11B shows the composition and flow of cluster #battery, while Figure 11C shows other clustering situations from 2015 to 2018, such as #big data, #automated metering infrastructure (AMI), #state estimation, #optimization, #distributed algorithm, and #communication.

Figure 11A shows the development process of cluster #SG. In the 2008–2009 stage, #SG only includes two subsets: SG and energy efficiency (EE). Among them, subset SG is transferred to the cluster in the stage 2010–2014 while EE is transferred to the other cluster #energy management. The subset of the new cluster #SG in the 2015–2018 phase has significantly increased. In addition to the orange SG stream, the subset also includes subsets who are transferred from the other cluster in the 2010–2014 phase. Subset demand response and electricity market transfers from cluster #energy management. Subset distribution network, distribution generation, renewable energy, and subset game theory is transferred from cluster #DG.

Similarly, Figure 11B shows the data flow of cluster #battery in 2015–2018, which is mainly composed of streams imported from 2010–2014. For example, vehicle to grid, privacy, EV, and smart meter are imported from the subset in the cluster #SG. Power grid, renewable energy resources, energy storage, and battery are imported from the subset in cluster #SG. Besides, the subset of cluster #energy management and the subset of cluster #delay wireless communication also converge to the cluster #battery in the last stage.

Figure 11C shows the inflow of subsets of the last few clusters, such as #communication network, #state estimation, #optimization, #AMI, and #big data. #big data consists of a lot of subsets: big data, cloud computing, data mining, IoT, machine learning, and power system. Among them, only IoT, power system, and cloud computing are transferred from phase 2010–2014, while the rest of the topics all involve the new subset. #AMI mostly flows from the previous phase, genetic algorithm, micro-grid (#DG), monitoring, WSN, AMI, energy efficiency (#energy management), the subset of the newly emerged subset is load management. #communication network consists of the quality of service (#delay) and communication network transfer in the former phase and also includes one subset of the newly emerged subset PLC. #distributed algorithm is a new cluster that includes two subsets: distributed algorithm and economic dispatch. #optimization includes four subsets: optimization (#battery), multi-agent system, control, and neural network. #state estimation consists mainly of a new subset including the state estimation, false data injection attack, CPS, cyber-attack, system, and smart substation (#smart substation).

SG's keywords at the three stages of development are more refined, in the first stage there are only two clusters, new clusters appear in the second phase, such as #battery, #delay, #based algorithm, #distributed generation, and #smart substation, together with the existing similar findings in section Author-Keywords Co-occurrence Analysis With Three Stages, the stage of SG has a great influence on the traditional power system and begins to change each link in power system research. In the third stage, these clusters are re-decomposed and combined to generate the new clusters #big data, #AMI, and #optimization. In addition to paying attention to the impact of the smart power grid on the traditional power grid, they also turn to intelligent equipment, data processing, and decision optimization.

## Conclusion

We applied different bibliometric methods and visualization tools to analyze the basic situation of China's SG, and tried to answer the questions raised in the first part by presenting bibliometric results.

Under the pressure of clean energy utilization and climate change, the development of SG has attracted the attention of various countries. This paper adopts a scientific visualization method to analyze 3,558 WOS literature records and uses scientometric techniques, such as co-authorship and co-occurrence, through which the status quo and trends of the SG field in China can be deeply understood.

1. The basics of SG in China: The publication volume presents an overall rising trend, but fluctuates after 2015. The research literature of Chinese scholars in SG is expected to continue to grow in the coming years. At the same time, from the perspective of discipline distribution, the literature in the SG field has obvious interdisciplinary characteristics. We found that the top 10 institutions in the SG field published half of the literature in the data set.
2. Cooperation on SG research in China. China has cooperative relations with 52 countries in the SG field, and the cooperation ratio among countries shows an increasing trend. The proportion of collaborative papers continues to rise and so does the proportion of the formation of several major partnerships.
3. Research hotspots of five productive institutions. SGCC, which has the largest number of published documents, is China's largest power grid service provider and is the main driver of SG development. It is committed to building an SG system based on power flow, information flow, and business flow of the entire power system. While increasing the consumption of renewable energy, SGCC plans to build a strong smart grid based on UHV, achieve coordinated development of power grids at all levels through information flow, and use it as a support to realize the integration of power generation, transmission, substation, distribution, power consumption, and dispatching business flow development. Its research topics are the most scattered and involve all aspects of SG research. In addition to SGCC, North China Electric Power University has a wide range of research hotspots, such as distributed power generation and micro-grid, demand response, EV, big data, and cloud computing. The research hotspots of Tsinghua University, CAS, and Shanghai Jiaotong University are relatively concentrated. In addition to demand response, Tsinghua University pays more attention to the cyber-physical system (CPS), power line communication (PLC), and EV. CAS pays more attention to power system planning and optimization, neural network, home energy management, and lithium-ion batteries. Shanghai Jiaotong University pays more attention to automatic generation control and big data.
4. Research hotspots and development trends. SG research has interdisciplinary characteristics, which is the integration of power system, engineering technology, and information communication technology. The research topics include engineering technology development, power system reconstruction, and information communication development. In terms of engineering technology, the development of battery technology has driven the development of EV and energy storage. Hot keywords include the battery, EV, energy storage, and lithium-ion battery. At the same time, the development of sensor technology provides technical support for data collection. Related keywords include IoT, sensors, smart meter, and AMI. Power system reconfiguration driven by SG thus affects all aspects of the power system research. Related keywords include micro-grid, distributed generation, smart substation, v2g, energy internet, and optimization. The development of information and communication technology is the foundation of the development of SG, and its main research hotspots include WSN, wireless communication, security, and big data. The development of China's SG research has evolved from a relatively single theme to the application of information and communication technology to integrate power system-wide research topics, including distributed power generation, smart grid, smart power transformation, smart dispatch, and demand response.

In the next step, further research into SG in China needs to focus on the following tasks: hardware design based on the IoT, network construction based on wireless transmission network, optimal dispatching of the power system based on demand response, improvement of energy efficiency, and the construction of regional and national smart grids.

### Strengths and Limitations

Quantitative analysis of a certain field or discipline based on bibliometric is a method that has gradually emerged in recent years. Although this method cannot be applied to accurately summarize the development of the discipline or field, it can find the basic characteristics and hotspots of the research of the discipline or field from a macro perspective. According to our search, this is the first bibliometric analysis of SG which uses alluvial diagrams to analyze the trend of keywords.

The research has provided valuable information for SG researchers, practitioners, and government institutions in China, meanwhile, the visualized map has provided valuable insight and an in-depth understanding of the key institutions, institutional and national cooperation, the current state of the research field, and the development trend.

The results of this study will be applied to (1) when government agencies and business organizations are formulating policies, consulting, and determining research cooperation; (2) when graduate students start to determine the current development situation of the gaps in understanding in the SG field; (3) when scholars make an attempt to understand the research hotspots in the SG field in China or need to discuss these topics with other scholars and seek potential cooperation.

There were some limitations in this study deserving our attention. First of all, our analysis is based on the analysis of the sample set, rather than the research results of the whole SG field in China. The SG field is developing rapidly and tends to be interdisciplinary. We are trying to extend it to the whole WOS library, however, the inclusion of the Emerging Sources Citation Index (ESCI) database starting in 2015, is different from the analysis period in this paper from 2008 to 2018, therefore this database was excluded in the analysis. In the subsequent research, the Scopus, Google Scholar, and other databases can be further taken into consideration for more comprehensive analysis in this field. Anyhow, the WOS database is the most comprehensive and widely used data at present, which is still meaningful for understanding the research status and development of the SG field in China. Secondly, this paper mainly considers SG research hotspots and its evolution in China from a macro perspective, but does not consider the relationship network of cooperation and co-citation between the authors, such as relationships regarding their common affiliations, academic supervisor-student relationships, and undoubtedly working experience is also important for understanding the development of SG research in China. Further research may consider the microscopic analysis of the authors and their cooperation.

## Data Availability Statement

All datasets generated for this study are included in the article/supplementary material.

## Author Contributions

CW, TL, and XD contributed to the conception and design of the study. XD organized the database. CW wrote the first draft of the manuscript. All authors contributed to manuscript revision and read and approved the submitted version.

## Conflict of Interest

The authors declare that the research was conducted in the absence of any commercial or financial relationships that could be construed as a potential conflict of interest.

## Footnotes

^1^Specific operational method and file format can refer to the website https://www.mapequation.org/apps/MapGenerator.html

## References

[B1] Baumeister T. (2010). Literature Review on Smart Grid Cyber Security. Simulation. Available online at: http://csdl.ics.hawaii.edu/techreports/10-11/10-11.pdf (accessed May 10, 2020).

[B2] Callon M. Courtial J. P. Turner W. A. Bauin S. (1983). From translations to problematic networks: an introduction to co-word analysis. Soc. Sci. Inform. 22, 191–235. 10.1177/053901883022002003

[B3] Chen C. (2013). Trajectories of Search, in Mapping Scientific Frontiers: The Quest for Knowledge Visualization, ed. C. Chen (London: Springer), 143–161. 10.1007/978-1-4471-5128-9_4

[B4] Chen C. (2017). Science mapping: a systematic review of the literature. J. Data Inform. Sci. 2, 1–40. 10.1515/jdis-2017-0006

[B5] Chen M. Mao S. Liu Y. (2014). Big data: a survey. Mob. Netw. Appl. 19, 171–209. 10.1007/s11036-013-0489-0

[B6] Cheng F. Chen J. (2012). Metal-air batteries: from oxygen reduction electrochemistry to cathode catalysts. Chem. Soc. Rev. 41, 2172–2192. 10.1039/c1cs15228a 22254234

[B7] Deng R. Yang Z. Chow M. Y. Chen J. (2015). A survey on demand response in smart grids: mathematical models and approaches. IEEE Trans. Industr. Inform. 11, 570–582. 10.1109/TII.2015.2414719

[B8] Ding Y. Rousseau R. Wolfram D. (2014). Measuring Scholarly Impact: Methods and Practice. Springer International Publishing. p. 346.

[B9] El-Hawary M. E. (2017). The Smart grid-state-of-the-art and future trends, in 2016 Eighteenth International Middle East Power Systems Conference (Cairo: MEPCON). 10.1109/MEPCON.2016.7836856

[B10] Fadaeenejad M. Saberian A. M. Fadaee M. Radzi M. A. M. Hizam H. Abkadir M. Z. A. (2014). The present and future of smart power grid in developing countries. Renew. Sustain. Energ. Rev. 29, 828–834. 10.1016/j.rser.2013.08.072

[B11] Gao J. Xiao Y. Liu J. Liang W. Chen C. L. P. (2012). A survey of communication/networking in smart grids. Future Gener. Comput. Syst. 28, 391–404. 10.1016/j.future.2011.04.014

[B12] Garfield E. (1979). Citation Indexing: Its Theory and Applications in Science, Technology, and Humanities. 1st Edn, ed. E. Garfield (New York, NY: John Wiley & Sons, Ltd).

[B13] Garfield E. (1983). Citation indexes for science a new dnenaion in documentation through associationof ideas. Science 1, 468–471.

[B14] Goran S. (2010). SSCI and its impact factors: a “prisoner's dilemma”? Eur. J. Mark. 44, 23–33. 10.1108/03090561011008583

[B15] Gungor V. C. Sahin D. Kocak T. Ergut S. Buccella C. Cecati C.. (2011). Smart grid technologies: communication technologies and standards. IEEE Trans. Ind. Inform. 7, 529–539. 10.1109/tii.2011.2166794

[B16] Guo Y. M. Huang Z. L. Guo J. Li H. Guo X. R. Nkeli M. J. (2019). Bibliometric analysis on smart cities research. Sustainability 11:3606. 10.3390/su11133606

[B17] He Q. (1999). Knowledge discovery through co-word analysis. Libr. Trends 48, 133–159.

[B18] Hirsch J. (2005). An index to quantify an individual's scientific research output. Proc. Natl. Acad. Sci. U.S.A. 102, 16569–16572. 10.1073/pnas.0507655102 16275915 PMC1283832

[B19] Hossain E. Khan I. Un-Noor F. Sikander S. S. Sunny M. S. H. (2019). Application of big data and machine learning in smart grid, and associated security concerns: a review. IEEE Access 7, 13960–13988. 10.1109/ACCESS.2019.2894819

[B20] Hossain M. S. Madlool N. A. Rahim N. A. Selvaraj J. Pandey A. K. Khan A. F. (2016). Role of smart grid in renewable energy: an overview. Renew. Sust. Energ. Rev. 60, 1168–1184. 10.1016/j.rser.2015.09.098

[B21] Hu M. Pang X. Zhou Z. (2013). Review recent progress in high-voltage lithium ion batteries. J. Power Sources 237, 229–242. 10.1016/j.jpowsour.2013.03.024

[B22] Kabalci Y. (2016). A survey on smart metering and smart grid communication. Renew. Sust. Energ. Rev. 57, 302–318. 10.1016/j.rser.2015.12.114

[B23] Lambiotte R. Rosvall M. (2012). Ranking and clustering of nodes in networks with smart teleportation. Phys. Rev. E 85:056107. 10.1103/PhysRevE.85.056107 23004821

[B24] Li F. Qiao W. Sun H. Wan H. Wang J. Xia Y.. (2010). Smart transmission grid: Vision and framework. IEEE Trans. Smart Grid 1, 168–177. 10.1109/TSG.2010.2053726

[B25] Lin J. Yu W. Zhang N. Yang X. Zhang H. Zhao W. (2017). A survey on internet of things: architecture, enabling technologies, security and privacy, and applications. IEEE Internet Things J. 4, 1125–1142. 10.1109/JIOT.2017.2683200

[B26] Liu J. Xiao Y. Li S. Liang W. Chen C. L. P. (2012). Cyber security and privacy issues in smart grids. IEEE Commun. Surv. Tutor. 14, 981–997. 10.1109/SURV.2011.122111.00145

[B27] Liu X. Bollen J. Nelson M. L. Van De Sompel H. (2005). Co-authorship networks in the digital library research community. Inform. Process. Manag. 41, 1462–1480. 10.1016/j.ipm.2005.03.012

[B28] Mahmood A. Javaid N. Razzaq S. (2015). A review of wireless communications for smart grid. Renew. Sust. Energ. Rev. 41, 248–260. 10.1016/j.rser.2014.08.036

[B29] Merigó J. M. Mulet-Forteza C. Valencia C. Lew A. A. (2019). Twenty years of tourism Geographies: a bibliometric overview. Tour. Geogr. 21, 881–910. 10.1080/14616688.2019.1666913

[B30] Montoya F. G. Montoya M. G. Gómez J. Manzano-Agugliaro F. Alameda-Hernández E. (2014). The research on energy in Spain: a scientometric approach. Renew. Sust. Energ. Rev. 29, 173–183. 10.1016/j.rser.2013.08.094

[B31] Mulet-Forteza C. Genovart-Balaguer J. Merigó J. M. Mauleon-Mendez E. (2019). Bibliometric structure of IJCHM in its 30 years. Int. J. Contemp. Hosp. Manag. 31, 4574–4604. 10.1108/IJCHM-10-2018-0828

[B32] National Development Reform Commission National Energy Administration (2015). Guiding Opinions on Promoting the Development of Smart Grid. National Development and Reform Commission. Available online at: http://www.nea.gov.cn/2015-07/07/c_134388049.htm (accessed July 15, 2020).

[B33] National Development Reform Commission National Energy Administration (2016). 13th Five-Year Plan for Electric Power Development (2016–2020). Available online at: http://zfxxgk.ndrc.gov.cn/web/iteminfo.jsp?id=398 (accessed July 20, 2020).

[B34] National Energy Administration (2010). Notice on the establishment of the National Smart Grid Standardization Overall Working Group. Available online at: http://www.nea.gov.cn/2011-10/12/c_131187554.htm (accessed July 20, 2020).

[B35] National Energy Administration Ministry of Justice (2020). Opinions on Speeding up the Establishment of Green Production and Consumption Regulations and Policy System. Available online at: https://www.ndrc.gov.cn/xxgk/zcfb/tz/202003/t20200317_1223470.html (accessed July 19, 2020).

[B36] Olawumi T. O. Chan D. W. M. (2018). A scientometric review of global research on sustainability and sustainable development. J. Clean. Prod. 183, 231–250. 10.1016/j.jclepro.2018.02.162

[B37] Olawumi T. O. Chan D. W. M. Wong J. K. W. (2017). Evolution in the intellectual structure of BIM research: a bibliometric analysis. J. Civil Eng. Manag. 23, 1060–1081. 10.3846/13923730.2017.1374301

[B38] Pan H. Hu Y. S. Chen L. (2013). Room-temperature stationary sodium-ion batteries for large-scale electric energy storage. Energ. Environ. Sci. 6, 2338–2360. 10.1039/c3ee40847g

[B39] Perianes-Rodriguez A. Waltman L. van Eck N. J. (2016). Constructing bibliometric networks: a comparison between full and fractional counting. J. Informetr. 10, 1178–1195. 10.1016/j.joi.2016.10.006

[B40] Ponce-Jara M. A. Ruiz E. Gil R. Sancristóbal E. Pérez-Molina C. Castro M. (2017). Smart grid: assessment of the past and present in developed and developing countries. Energy Strategy Rev. 18, 38–52. 10.1016/j.esr.2017.09.011

[B41] Rahimi-Eichi H. Ojha U. Baronti F. Chow M. Y. (2013). Battery management system: an overview of its application in the smart grid and electric vehicles. IEEE Ind. Electron. Mag. 7, 4–16. 10.1109/MIE.2013.2250351

[B42] Rahman A. I. M. J. Guns R. Rousseau R. Engels T. C. E. (2017). Cognitive distances between evaluators and evaluees in research evaluation: a comparison between three informetric methods at the journal and subject category aggregation level. Front. Res. Metr. Anal. 2:6. 10.3389/frma.2017.00006

[B43] Rosvall M. Bergstrom C. T. (2008). Maps of random walks on complex networks reveal community structure. Proc. Natl. Acad. Sci. U.S.A. 105, 1118–1123. 10.1073/pnas.0706851105 18216267 PMC2234100

[B44] Rosvall M. Bergstrom C. T. (2010). Mapping change in large networks. PLoS ONE 5:e8694. 10.1371/journal.pone.0008694 20111700 PMC2811724

[B45] Shi Y. Liu X. (2019). Research on the literature of green building based on the web of science: a scientometric analysis in citespace (2002–2018). Sustainability 11:3716. 10.3390/su11133716

[B46] Strasser T. Andrén F. Kathan J. Cecati C. Buccella C. Siano P.. (2015). A review of architectures and concepts for intelligence in future electric energy systems. IEEE Trans. Industr. Electron. 62, 2424–2438. 10.1109/TIE.2014.2361486

[B47] Su W. Eichi H. Zeng W. Chow M. Y. (2012). A survey on the electrification of transportation in a smart grid environment. IEEE Trans. Industr. Inform. 8, 1–10. 10.1109/TII.2011.2172454

[B48] Subramanyam K. (1983). Bibliometric studies of research collaboration: a review. J. Inform. Sci. 6, 33–38. 10.1177/016555158300600105

[B49] Sun Y. Zhao L. Pan H. Lu X. Gu L. Hu Y. S.. (2013). Direct atomic-scale confirmation of three-phase storage mechanism in Li4Ti5 O12 anodes for room-temperature sodium-ion batteries. Nat. Commun. 4:1870. 10.1038/ncomms2878 23695664

[B50] Tan X. Li Q. Wang H. (2013). Advances and trends of energy storage technology in Microgrid. Int. J. Electr. Power Energy Syst. 44, 179–191. 10.1016/j.ijepes.2012.07.015

[B51] Tsui K. M. Chan S. C. (2012). Demand response optimization for smart home scheduling under real-time pricing. IEEE Trans. Smart Grid 3, 1812–1821. 10.1109/TSG.2012.2218835

[B52] Tu C. He X. Shuai Z. Jiang F. (2017). Big data issues in smart grid – a review. Renew. Sust. Energ. Rev. 79, 1099–1107. 10.1016/j.rser.2017.05.134

[B53] Tuballa M. L. Abundo M. L. (2016). A review of the development of Smart Grid technologies. Renew. Sust. Energ. Rev. 59, 710–725. 10.1016/j.rser.2016.01.011

[B54] Usman A. Shami S. H. (2013). Evolution of communication technologies for smart grid applications. Renew. Sust. Energ. Rev. 19, 191–199. 10.1016/j.rser.2012.11.002

[B55] van Eck N. J. Waltman L. (2010). Software survey: VOSviewer, a computer program for bibliometric mapping. Scientometrics 84, 523–538. 10.1007/s11192-009-0146-3 20585380 PMC2883932

[B56] Varaiya P. P. Wu F. F. Bialek J. W. (2011). Smart operation of smart grid: risk-limiting dispatch: ways of managing energy systems without endangering reliability, while utilizing many intermittent resources, are discussed in this paper. Proc. IEEE 99, 40–57. 10.1109/JPROC.2010.2080250

[B57] Wang Q. Zhang C. Ding Y. Xydis G. Wang J. Østergaard J. (2015). Review of real-time electricity markets for integrating distributed energy resources and demand response. Appl. Energy 138, 695–706. 10.1016/j.apenergy.2014.10.048

[B58] Wang Y. (2010). Research framework of technical standard system of strong & smart grid. Automa. Electr. Power Syst. 34, 1–6 [in Chinese].

[B59] Wang Y. Yu X. Xu S. Bai J. Xiao R. Hu Y. S.. (2013). A zero-strain layered metal oxide as the negative electrode for long-life sodium-ion batteries. Nat. Commun. 4:2365. 10.1038/ncomms3858 23978932

[B60] Wu C. Mohsenian-Rad H. Huang J. (2012). Vehicle-to-aggregator interaction game. IEEE Trans. Smart Grid 3, 434–442. 10.1109/TSG.2011.216641430962617

[B61] Xiao J. Gu W. Wang C. Li F. (2012). Distribution system security region: definition, model and security assessment. IET Gener. Transm. Distrib. 6, 1029–1035. 10.1049/iet-gtd.2011.0767

[B62] Yu D. He X. (2020). A bibliometric study for DEA applied to energy efficiency: trends and future challenges. Appl. Energy 268:115048. 10.1016/j.apenergy.2020.115048

[B63] Yu D. Xu Z. Wang X. (2020). Bibliometric analysis of support vector machines research trend: a case study in China. Int. J. Mach. Learn. Cybern. 11, 715–728. 10.1007/s13042-019-01028-y

[B64] Yu W. Liang F. He X. Hatcher W. G. Lu C. Lin J.. (2017). A survey on the edge computing for the internet of things. IEEE Access 6, 6900–6919. 10.1109/ACCESS.2017.2778504

[B65] Yu Y. Yang J. Chen B. (2012). The smart grids in China-a review. Energies 5, 1321–1338. 10.3390/en5051321

[B66] Yuan J. Hu Z. (2011). Low carbon electricity development in China - an IRSP perspective based on super smart grid. Renew. Sust. Energ. Rev. 15, 2707–2713. 10.1016/j.rser.2011.02.033

[B67] Yuan J. Shen J. Pan L. Zhao C. Kang J. (2014). Smart grids in China. Renew. Sust. Energ. Rev. 37, 896–906. 10.1016/j.rser.2014.05.051

[B68] Zhang D. Han X. Deng C. (2018). Review on the research and practice of deep learning and reinforcement learning in smart grids. CSEE J. Power Energy Syst. 4, 362–370. 10.17775/CSEEJPES.2018.00520

[B69] Zhang Y. Yu R. Nekovee M. Liu Y. Xie S. Gjessing S. (2012). Cognitive machine-to-machine communications: visions and potentials for the smart grid. IEEE Netw. 26, 6–13. 10.1109/MNET.2012.6201210

[B70] Zhao B. Ran R. Liu M. Shao Z. (2015). A comprehensive review of Li4Ti5O12-based electrodes for lithium-ion batteries: the latest advancements and future perspectives. Mater. Sci. Eng. R Rep. 98, 1–71. 10.1016/j.mser.2015.10.001

[B71] Zhong H. Xie L. Xia Q. (2013). Coupon incentive-based demand response: theory and case study. IEEE Trans. Power Syst. 28, 1266–1276. 10.1109/TPWRS.2012.2218665

[B72] Zhong Q. C. Nguyen P. L. Ma Z. Sheng W. (2014). Self-synchronized synchronverters: inverters without a dedicated synchronization unit. IEEE Trans. Power Electron. 29, 617–630. 10.1109/TPEL.2013.2258684

[B73] Zhou K. Fu C. Yang S. (2016). Big data driven smart energy management: From big data to big insights. Renew. Sust. Energ. Rev. 56, 215–225. 10.1016/j.rser.2015.11.050

[B74] Zhu J. Song L. J. Zhu L. Johnson R. E. (2019). Visualizing the landscape and evolution of leadership research. Leadersh. Q. 30, 215–232. 10.1016/j.leaqua.2018.06.003

